# Meta-analysis of AI posture recognition for work-related musculoskeletal disorders prevention in occupational activities: comparison between untransformed, logit and Freeman-Tukey transformed performance

**DOI:** 10.3389/frai.2026.1933096

**Published:** 2026-10-07

**Authors:** Philippe Gorce, Julien Jacquier-Bret

**Affiliations:** 1 University of Toulon, Toulon, France; 2 International Institute of Biomechanics and Occupational Ergonomics, Hyères, France

**Keywords:** accuracy, deep learning, ergonomic assessment, F1 score, machine learning, precision, sensitivity, specificity

## Abstract

**Introduction:**

The objective of this systematic review and meta-analysis was to evaluate the performance of artificial intelligence (AI) posture recognition systems in preventing the occurrence of work-related musculoskeletal disorders (WMSDs) across all occupational activities, following the PRISMA (Preferred Reporting Items for Systematic Reviews and Meta-Analyses) guidelines.

**Methods:**

During May 2026, six databases were screened without publication date restrictions. Selected studies had to evaluate the performance of a posture recognition system using a machine learning (ML) or deep learning (DL) approach to assess the risk level of WMSD development using at least one of the following metrics: accuracy, specificity, sensitivity, precision, and F1 score. Risk of bias, heterogeneity, sensitivity, publication bias, and certainty of evidence analyses were conducted in the meta-analysis. Logit and Freeman-Tukey transformations were applied to the data before pooling and results were compared to those obtained from the untransformed. A meta-analysis was performed by performance parameter and by transformation. Four subgroup analyses were performed: comparison of AI methods, sensor types, professional activities, and ergonomic tools used, using pairwise mixed-effects meta-regressions.

**Results:**

Among the 1,071 unique entries, 26 studies were included. The logit transformation most closely approximated a normal distribution for all performance metrics without affecting heterogeneity, which was high (I^2^ > 90%) in all analyses. Overall performance ranged from 85 to 95% for the five performance metrics. DL significantly outperformed ML by 4 to 16% for specificity, sensitivity, and accuracy, regardless of the transformation (*p* ≤ 0.05). Hybrid solutions demonstrated higher sensitivity and accuracy (> 99%, *p* < 0.05) with low heterogeneity (I^2^ < 30%) regardless of the transformation. Occupational activity had a limited influence on overall performance, and no assessment tool consistently outperformed all others across all five performance parameters.

**Conclusion:**

In conclusion, the logit transformation consistently improved the statistical properties of the data and AI approach, type of sensors, occupational activity, and ergonomic risk assessment tool all significantly influenced the reported performance metrics. However, findings should be interpreted with caution and prudence is required regarding the deployment of the systems under real-world conditions due to the very low level of evidence.

**Systematic review registration:**

https://www.crd.york.ac.uk/PROSPERO/home, PROSPERO, n°: CRD420261432405.

## 1 Introduction

Reducing work-related musculoskeletal disorders (WMSDs) is a major worldwide challenge. These disorders manifest as inflammation and degeneration of various tissues in the body—including bone, muscle, joint, and nerve tissues—which, with prolonged exposure, can lead to painful conditions or syndromes (Punnett and Wegman, 2004; Jacquier-Bret and Gorce, 2025). More than 20% of the population suffers from musculoskeletal disorders [over 1.71 billion people in 2019 (Cieza et al., 2021)]. In the workplace, the prevalence of WMSDs is high across all sectors [healthcare (Jacquier-Bret and Gorce, 2023; Gorce and Jacquier-Bret, 2025a), manufacturing (Govaerts et al., 2021; He et al., 2023), the service sector (Mohammadian et al., 2025; Tahernejad et al., 2024), etc.]. They are a leading cause of disability and absenteeism, resulting in significant economic losses (EU-OSHA, 2019; U.S. Bureau of Labor Statistics, 2020).

Maintaining and repeated awkward postures during work activities is one of the main factors responsible for the occurrence and exacerbation of WMSDs (Gorce and Jacquier-bret, 2024; Bispo et al., 2022; da Costa and Vieira, 2010). Several ergonomic approaches have been used to assess the risk of WMSDs associated with work postures. Early approaches relied on the presence of postures already identified as risky to assess a workstation (Tolera et al., 2025). Working overhead, twisting or bending postures, or working on one’s knees are situations recognized as risky (Punnett and Wegman, 2004; Bernard, 1997). The Occupational Safety and Health Administration (OSHA) drew on these awkward postures and combined them with other workplace risk factors such as the force exerted, repetitiveness, duration of exposure, and other specific and organizational factors (Occupational Safety and Health Administration (OSHA), 2024). The more numerous, intense, and prolonged these factors are, the higher the risk associated with the workstation. Other approaches have objectified the assessment of WMSD risk using a score derived from several factors. Based on a pre-established scale, this score indicates the level of risk and the intervention to be implemented. The Ovako Working Posture Analysing System (OWAS) is a postural observation method developed to assess risk based on the work postures adopted (Karhu et al., 1977). The method assesses the posture of the back, arms, and legs based on a predefined list and includes the load being handled. The ergonomic tools RULA [Rapid Upper Limb Assessment (Mc Atamney and Nigel Corlett, 1993)] and REBA [Rapid Entire Limb Assessment (Hignett and Mc Atamney, 2000)] have expanded upon the posture assessments proposed by OWAS. They have incorporated objective measurements of several biomechanical angles to establish scores for the cervical and lumbar regions, as well as the upper and lower limbs (further developed in REBA), along with task-related information such as load, grip, and activity. Finally, other approaches have been developed for specific activities, such as load-carrying using the Revised National Institute for Occupational Safety and Health (NIOSH) Lifting Equation (Waters et al., 1993) or the Hand Activity Level (Latko et al., 1997) to assess risks associated with repetitive upper-limb movements.

However, these observation tools focus on a specific posture at a given moment or on a sequence of postures captured at regular intervals. Furthermore, they require the involvement of a specialized operator and processing time (Jacquier-Bret and Gorce, 2024; Gómez-Galán et al., 2020). Consequently, feedback is delayed and does not provide a comprehensive view of the risks associated with a work activity that extends over time. For a more realistic assessment of the risks of developing WMSDs due to awkward postures, it is essential to collect data continuously to cover the entire duration of the work activity. This process inevitably generates a larger volume of data, requiring greater resources for its processing. In this context, artificial intelligence (AI) offers significant advantages, particularly due to its ability to process large amounts of data with complex patterns. Its use has grown with the advent of Industry 4.0, which has fostered the implementation of automated equipment and digital solutions aimed at increasing productivity while ensuring workplace safety.

Whether using non-wearable vision-based sensors (Wang et al., 2021) or wearable sensors integrated into work clothing or directly attached to body parts (Naranjo et al., 2025), it is now possible to continuously monitor operators’ activity in real time. In the context of WMSD prevention, AI-based devices are primarily designed to detect the risk factors to which workers are exposed—particularly postures—to enable effective ergonomic interventions (Ding et al., 2023; Demissie et al., 2024). Recent technological advances in various types of sensors and communication protocols provide continuous access to a wide range of information, whether physiological, muscular, or movement-related (De Fazio et al., 2023). Thanks to these databases and advanced algorithms, AI is able to autonomously classify individuals’ routine activities within defined contexts. Depending on the task and the technologies used, this can range from simple event detection to the identification and classification of postures. Numerous studies have proposed posture recognition solutions that use one of two AI methods: machine learning (ML) and deep learning (DL). ML methods rely on large databases to train various algorithms, which are then used to recognize and classify postures. Among the most common are logistic regression (Prisco et al., 2024), the k-nearest neighbors (KNN; Suárez Sánchez et al., 2016) algorithm, decision trees (DT; Su et al., 2023), and support vector machines (SVM; Matos et al., 2024). ML methods are able to automatically extract, without human intervention, the features necessary for posture detection from raw data (LeCun et al., 2015). Long short-term memory (LSTM; Zhou et al., 2022), gated recurrent units (GRU; Antwi-Afari et al., 2022), and convolutional neural networks (CNN; Young et al., 2023) have been used most frequently.

One of the key issues associated with posture-based WMSD prevention systems is the system’s ability to correctly recognize and classify these postures. Thus, when designing the algorithms, the authors evaluate performance using a confusion matrix that includes four criteria: true positives, false positives, true negatives, and false negatives. Based on these criteria, five performance metrics are typically computed: accuracy, precision, specificity, sensitivity, and the F1 score (Santos et al., 2019; Zhang et al., 2025). These metrics capture various aspects of the posture recognition system’s performance and WMSD risk assessment, including the proportion of postures that are actually at risk and the rate of false alarms. Given the growing use of AI and the wide variety of available solutions, there are several effective posture recognition algorithms for preventing WMSDs. However, these algorithms are directly linked to the working conditions of the specific occupational activities in question. Several authors have published literature reviews to summarize advances in this field (Çakit and Karwowski, 2025; Shakerian et al., 2025; Yang et al., 2024; Donisi et al., 2022). Based on between 25 and 130 studies, these works have highlighted research gaps and proposed directions for future applications related to WMSD management, whether for an AI method or a category of sensors. However, although some reviews reported a few performance metrics, performance data are often missing or rarely reported in detail, even though they are crucial to the viability of these systems. To our knowledge, only the recent study by Gorce and Jacquier-Bret (2026a) has provided a summary of the five performance metrics by occupational activity. However, the authors did not apply the full meta-analysis procedure to provide pooled values indicating the current trend in performance levels achieved by posture recognition systems. To our knowledge, there is no systematic review with a meta-analysis examining the performance of AI-based methods for assessing the WMSD risk across all occupational activities.

In this context, the objective of this study was to evaluate the performance of posture detection systems designed to prevent WMSDs in the workplace, using a meta-analysis conducted in accordance with the PRISMA guidelines (Preferred Reporting Items for Systematic Reviews and Meta-Analyses (Page et al., 2021)). Adherence to these guidelines addresses issues related to the robustness and level of evidence required for generalizing the results, applying them, and formulating recommendations. The meta-analysis was conducted for the following five performance metrics: accuracy, specificity, sensitivity, precision, and F1 score. In-depth analyses were performed to explore possible causes of heterogeneity by considering the two AI approaches, occupational activities, different sensor categories, and the various ergonomic tools used to define the level of WMSD risk. Since the performance metrics are binomial proportions ranging from 0 to 1, they are often skewed toward the extreme values and do not satisfy the normality assumptions of the random-effects models used in meta-analysis. Applying a transformation before data synthesis is a way to stabilize the variance and approximate a normal distribution model. One of the original aspects of this study was to compare the untransformed pooled values with those obtained using two transformations commonly used to handle extreme proportions—namely, the logit transformation (Seiffert et al., 2024) and the Freeman-Tukey double arcsine transformation (Freeman and Tukey, 1950). The objective was to provide evidence to guide future research and help industry leaders and policymakers improve workplace safety and quality of life.

## 2 Methods

### 2.1 Study design

This study is a systematic review and meta-analysis conducted in accordance with the PRISMA (Preferred Reporting Items for Systematic Reviews and Meta-Analyses) guidelines (Page et al., 2021; Harris et al., 2014; Moher et al., 2009). The objective was to evaluate the performance of AI-based posture recognition systems in preventing the occurrence of WMSDs during occupational activities. To be included, studies had to be original research, assess the level of WMSD risk using an ergonomic tool or a classification of postures defined as safe and unsafe via a ML or DL method, and measure performance using at least one of the following five metrics: accuracy, sensitivity, specificity, precision, or F1 score.

### 2.2 Search strategy

Six databases were screened in May 2026 to identify relevant studies, with no restriction on publication date: Academia, Google Scholar, IEEE Xplore, PubMed/MedLine, ScienceDirect, and Science.gov. Table 1 presents the combination of keywords linked by the Boolean operators AND and OR used in the search. Given the limit of 8 Boolean operators imposed by the ScienceDirect search engine, the combination had to be adjusted. Depending on the database, a filter was applied to preselect studies that met the selection criteria (see Table 1 legend).

**Table 1:** Keyword combination for each database.

Database	Keyword combination
Academia, Google Scholar, IEEE Xplore, PubMed/MedLine, Science.gov	posture AND (“artificial intelligence” OR AI) AND (“work-related musculoskeletal disorders” OR “WMSDs”) AND (accuracy OR precision OR “F1 score” OR F1-score OR specificity OR sensitivity)
ScienceDirect	posture AND (“artificial intelligence”) AND (“work-related musculoskeletal disorders” OR WMSDs) AND (accuracy OR precision OR sensitivity OR specificity OR “F1 score”)

For Academia, the “English” filter was used. For Google Scholar, the “journal article” filter was used; for IEEE Xplore, the “Journal” filter was used; for ScienceDirect, the “Research articles” filter was used. No filters were applied for PubMed/MedLine and Science.gov.

### 2.3 Selection process

The selection of studies was conducted in two successive stages: selection from databases, followed by snowball sampling based on the reference lists of the evaluated articles.

During the first stage, all items identified in each database were compiled into a single Excel file (Microsoft® Office Excel 2019, WA, United States). Each row represented a result and contained the authors’ names, year of publication, title, and abstract. Excel’s search and duplicate removal function was used to remove multiple entries for the same article. Using the list of unique entries, two reviewers (PG and JJB) independently assessed the titles and abstracts to select articles that met the inclusion and exclusion criteria. The lists of articles selected by each reviewer were compared to establish the list of articles to be retrieved for the next step of the process. Each full-text article was then evaluated separately by each reviewer to determine the final list of articles included in the meta-analysis. At each step, discrepancies were resolved through discussion to reach a consensus, following a re-reading and re-evaluation of the article if necessary.

The second stage began with the evaluation of the full-text articles. Each reviewer identified additional potentially relevant studies in the reference list (using the “snowball” selection method). Each of these articles was then evaluated independently by both reviewers. If it met the selection criteria, the article was manually added to the previous list after consensus was reached between the two reviewers.

### 2.4 Inclusion/exclusion criteria

To be included, a study had to meet several criteria. First, the authors had to present an AI-based posture recognition method—i.e., one using ML or DL approach—in the context of occupational activities. This method had to aim to assess the risk of WMSDs based on the postures studied, using either a quantified risk assessment (a scale derived from ergonomic assessment tools) or a predefined list of high-risk postures. Second, authors were required to quantitatively evaluate the performance of their AI solution using at least one of the following metrics: accuracy, precision, specificity, sensitivity, and F1 score. Finally, the studies had to be original research, published in English, and peer-reviewed to ensure a sufficient level of quality.

The following exclusion criteria were applied: (1) the study was a conference paper, a book or chapter, a review, a report, a case study, or a case report; (2) the study was not written in English; (3) the level of ergonomic risk for WMSDs was not assessed qualitatively or quantitatively; (4) the process for characterizing postures was not sufficiently precise; (5) methodological details were insufficient; (6) the validation procedure was not clearly presented (data split between training and testing); (7) performance parameters were not available.

### 2.5 Risk of bias assessment

The risk of bias for each diagnostic test accuracy study (DTAS) was assessed using the PROBAST tool [Prediction Model Study Risk of Bias Assessment Tool (Wolff et al., 2019)]. The PROBAST tool evaluates 20 methodological points in the form of a question to be answered using one of the following five responses: “yes,” “probably yes,” “probably no,” “no,” or “information missing.” The questions were formulated such that a “yes” response indicates the absence of bias. The 20 questions cover four domains of each DTAS: participants (2 questions), predictors (3 questions), outcomes (6 questions), and analysis (9 questions). Based on the responses to the questions, a level of risk of bias is assigned to each domain: “low” if all responses were “yes” or “probably yes,” “high” if at least one question received a “no” or “probably no” response, or “moderate” if at least one response was “information missing” and the other responses were “yes” or “probably yes.” Based on the risk level assigned to each domain, a judgment is conducted to determine the overall risk of bias: “low” if all domains had a low risk of bias; “high” if at least one domain had a high risk of bias; “moderate” in all other cases. Since the validation of an AI method is an essential step for its use in real-world conditions, the absence of internal or external validation consistently led to a high risk of bias assessment (Gorce and Jacquier-Bret, 2025b). The risk level for each domain and the overall judgment were presented in the form of a traffic-light plot (McGuinness and Higgins, 2021).

### 2.6 Data extraction

The data from each study were extracted separately by the two reviewers (PG and JJB). Four categories were documented for each study: general information (first authors, year of publication, and occupational activity), information related to the WMSD assessment (work tasks performed, postures adopted, and ergonomic assessment tool), the methodology used (nature and placement of sensors, number of subjects tested, number of features measured, chosen AI method and algorithm, as well as the validation procedure), and the values of the performance metrics, i.e., accuracy, specificity, sensitivity, precision, and F1 score. The data were organized and presented in the form of summary tables. The content was validated by both reviewers, who reached a consensus in cases of disagreement during data extraction.

Each metric evaluates a specific aspect of the AI posture recognition solution. They are derived from the four pieces of information contained in the confusion matrix (Figure 1): correct positive predictions (true positives, TP), correct negative predictions (true negatives, TN), incorrect positive predictions (false positives, FP), and incorrect negative predictions (false negatives, FN).

**Figure 1:**
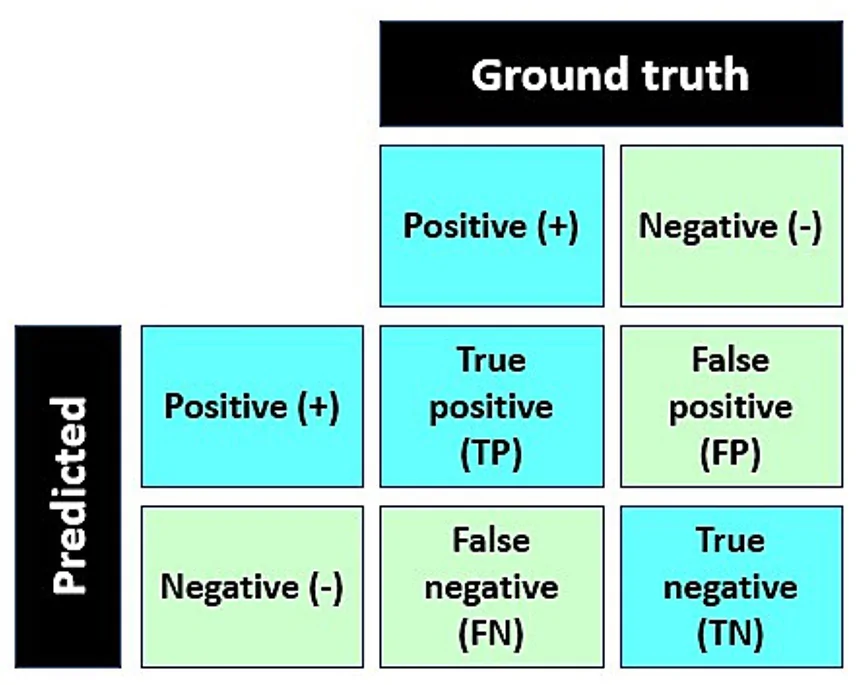
Confusion matrix used to compute the performance metrics.

Accuracy is the proportion of all predictions correctly classified by the model, including both positive and negative cases. The formula is:

$$\text{Accuracy}=\frac{\mathrm{TP}+\mathrm{TN}}{\mathrm{TP}+\mathrm{TN}+\mathrm{FP}+\mathrm{FN}}$$

Specificity (true negative rate) is the model’s ability to correctly identify cases that are actually negative. High specificity indicates a low proportion of false positives. The formula is:

$$\text{Specificity}=\frac{\mathrm{TN}}{\mathrm{TN}+\mathrm{FP}}$$

Sensitivity (true positive rate) is the model’s ability to correctly identify cases that are actually positive. High sensitivity indicates a low proportion of false negatives. The formula is:

$$\text{Sensitivity}=\frac{\mathrm{TP}}{\mathrm{TP}+\mathrm{FN}}$$

Precision (positive predictive value) is the proportion of positive predictions made by the model that are actually correct. This metric reflects the reliability of positive classifications. The formula is:

$$\text{Precision}=\frac{\mathrm{TP}}{\mathrm{TP}+\mathrm{FP}}$$

F1 score is the harmonic mean of precision and sensitivity. It provides a balanced measure of performance when one wishes to simultaneously account for false positives and false negatives, particularly in the presence of imbalanced classes. The formula is:

$$F1\;\text{score}=2\times \frac{\text{sensitivity}\times \text{precision}}{\text{sensitivity}+\text{precision}}$$

### 2.7 Statistical analysis

To conduct the meta-analysis, a random-effects model estimated using the Restricted Maximum Likelihood (REML) method was selected, given the expected high heterogeneity. This model assumes that the observed effect sizes are normally distributed around their true effects, while the true effects themselves are assumed to follow a normal distribution across studies. However, the five indicators that characterize the performance of AI solutions are ratios ranging from 0 to 1. Since the objective is to maximize them, their distribution is often skewed and does not satisfy the normality assumption. Then, it is recommended to transform the data before their aggregation. This procedure is used, on the one hand, to stabilize the variance and, on the other hand, to bring the distribution closer to a normal distribution (Barendregt et al., 2013), thereby better satisfying the statistical assumptions of meta-analysis models. The Freeman-Tukey transformation (Freeman and Tukey, 1950) is based on a double arcsine transformation, which is particularly effective at handling extreme proportions and issues related to small samples. The main limitation is the introduction of a back-transformation bias in some situations. The logit transformation is increasingly used in meta-analyses (Seiffert et al., 2024). It maps proportions from the bounded interval [0,1] to the real line, reducing skewness and facilitating the application of random-effects models. Although it is well-suited for handling proportions close to the bounds, its main limitation lies in the need to perform a continuity correction because the transformation is undefined for proportions exactly equal to 0 or 1.

Prior to pooling the meta-analysis, the proportions for the five performance parameters were transformed separately using the logit transformation and the Freeman-Tukey (FT) transformation. The respective formulas used were:

$$\text{logit}(p)=L=\ln(\frac{p}{1-p})$$

and

$$\text{FT}(p)=\beta =\frac{1}{2}(\arcsin(\sqrt{\frac{x}{n+1}})+\arcsin(\sqrt{\frac{x+1}{n+1}}))$$

where *x* is the number of favorable cases, *n* is the sample size, *p* is the performance proportion (ratio of *x* to *n*), and *ln* is the natural logarithm.

When *p* = 0 or *p* = 1, the logit transformation is mathematically impossible. In this case, a Haldane-Anscombe continuity correction (*p_(HA)_*) (Haldane, 1940; Anscombe, 1956) was applied to recompute *p* before performing the logit transformation:

$$p_{\left(\mathrm{HA}\right)}=\frac{x+0.5}{n+1}$$

where *x* is the number of favorable cases and *n* is the sample size.

The standard error (SE) corresponding to each transformation was computed using the following respective formulas:

$$SE_{\text{logit}}(p)=\sqrt{\frac{1}{x}+\frac{1}{n-x}}$$

$$SE_{\text{FT}}(p)=\sqrt{\frac{1}{4n+2}}$$

where *x* is the number of favorable cases and *n* is the sample size. The standard error (*SE*) was also calculated for the untransformed proportions using the following formula:

$$SE_{\mathrm{UT}}(p)=\sqrt{\frac{p(1-p)}{n}}$$

where *p* is the performance proportion and *n* is the sample size.

Once the transformations were performed, a meta-analysis by performance metric was conducted. The untransformed values, as well as those resulting from the two transformations, were successively pooled using a random-effects model due to the high heterogeneity. Inter-study variability (τ^2^) was estimated using the inverse variance method with the REML model. Heterogeneity was assessed using Cochran’s Q test (significance threshold set at 10%) and the I^2^ statistic (Cochrane Training, 2021). The I^2^ value was used to classify heterogeneity as low (0–40%), moderate (30–60%), substantial (50–90%), and high (75–100%). For each analysis, the results were presented as forest plots showing pooled values and 95% confidence intervals.

In-depth analyses were conducted to explore the causes of heterogeneity. Four subgroup analyses were performed: the effect of sensor family, AI method, occupational activity, and the ergonomic method used to define the WMSD risk level. The differences among the various factors in each analysis were compared using a pairwise mixed-effects meta-regression. The robustness of the results was tested using a sensitivity analysis involving the successive exclusion of one factor at a time. Visual analysis of the funnel plot and Egger’s test (Egger et al., 1997) were used to examine data asymmetry and the risk of publication bias.

To interpret and compare the pooled performance results, all proportions were converted back (*pBT*) to their original scale using the following equations, corresponding to each transformation:

$$\mathrm{pB}T_{\text{logit}}=\frac{e^{L}}{1+e^{L}}$$

$$\mathrm{pB}T_{\text{FT}}={\left(\sin(\beta )\right)}^{2}$$

where *L* represents the value on the logit scale and *β* represents the value on the Freeman-Tukey scale.

In order to compare the two transformations with the untransformed values, an analysis of several indicators of normality, symmetry, residual diagnostics, and heteroscedasticity was conducted to identify the most appropriate transformation for each metric. Four measures of normality were considered: the QQ-plot correlation, the Shapiro–Wilk test, skewness, and kurtosis. The QQ-plot correlation measures how closely the observed distribution approximates a theoretical normal distribution. The closer this correlation is to 1, the more the distribution is consistent with the assumption of normality (Wilk and Gnanadesikan, 1968). The Shapiro–Wilk test detects deviations from normality. The closer a transformation brings the Shapiro–Wilk W value to 1, the closer the approximation is to a normal distribution (Shapiro and Wilk, 1955). Skewness quantifies the asymmetry of the distribution, and kurtosis measures the weight of the distribution’s tails. The closer their respective values are to zero, the more normal the distribution (Kim, 2013). Additional parameters have been added. Studentized residuals help identify studies exhibiting atypical behavior relative to the model. Values greater than ±3 are generally considered potentially influential observations. A good transformation should reduce the number of extreme residuals (Viechtbauer and Cheung, 2010). Finally, I^2^ represents the proportion of variability explained by between-study heterogeneity. A transformation that reduces I^2^ generally indicates better stabilization of the variance (Higgins and Thompson, 2002).

All analyses were performed using the JASP (JASP Team, v0.96, Amsterdam, Netherlands) and R (R Core Team, v4.6.0, Vienna, Austria) software with a significance threshold set at 5%.

### 2.8 Certainty of evidence

The GRADE (Grade of Recommendations Assessment, Development and Evaluation) tool (Guyatt et al., 2011) was used to assess the quality of evidence for each transformation. Each of the five domains—risk of bias, indirectness of evidence, inconsistency of results, imprecision of results, and publication bias—was scored as “serious” or “not serious” based on the information they contained. From this assessment, a final judgment was made on a four-level scale: high, moderate, low, and very low. The rule was as follows: a DTAS starts with a high level of evidence, and a downgrade was applied for each domain where the risk was considered “serious.” The entire assessment was conducted independently by the two reviewers (PG and JJB). The results were compared, and any discrepancies were resolved by consensus. The final assessment was presented with summary tables.

### 2.9 Protocol registration

The protocol was registered in the PROSPERO database under the number CRD420261432405.

## 3 Results

### 3.1 Search results

The scan of the 6 databases identified 1,214 studies, 143 of which were duplicates. After removing the duplicates, 914 of the 1,071 unique studies were excluded based on their titles and abstracts. The main reasons were: the study was not an original, peer-reviewed research study; the assessment of WMSDs was absent or insufficiently detailed; the AI method used was not explicit enough to classify the study. From the remaining 157 studies, 5 reports could not be retrieved, and 129 were excluded after reviewing the full text—either because an ergonomic evaluation tool was not included, because the study did not report the performance metric values of the AI method used, or because information regarding the training/testing data split was not specified. At the end of this phase, 23 studies were retained.

During the full-text evaluation phase, 15 studies were identified through reference screening. Twelve were excluded after evaluation of their full texts, and 3 were manually added to the 23 previously selected studies. Thus, this systematic review included 26 studies. Figure 2 details the entire selection process.

**Figure 2:**
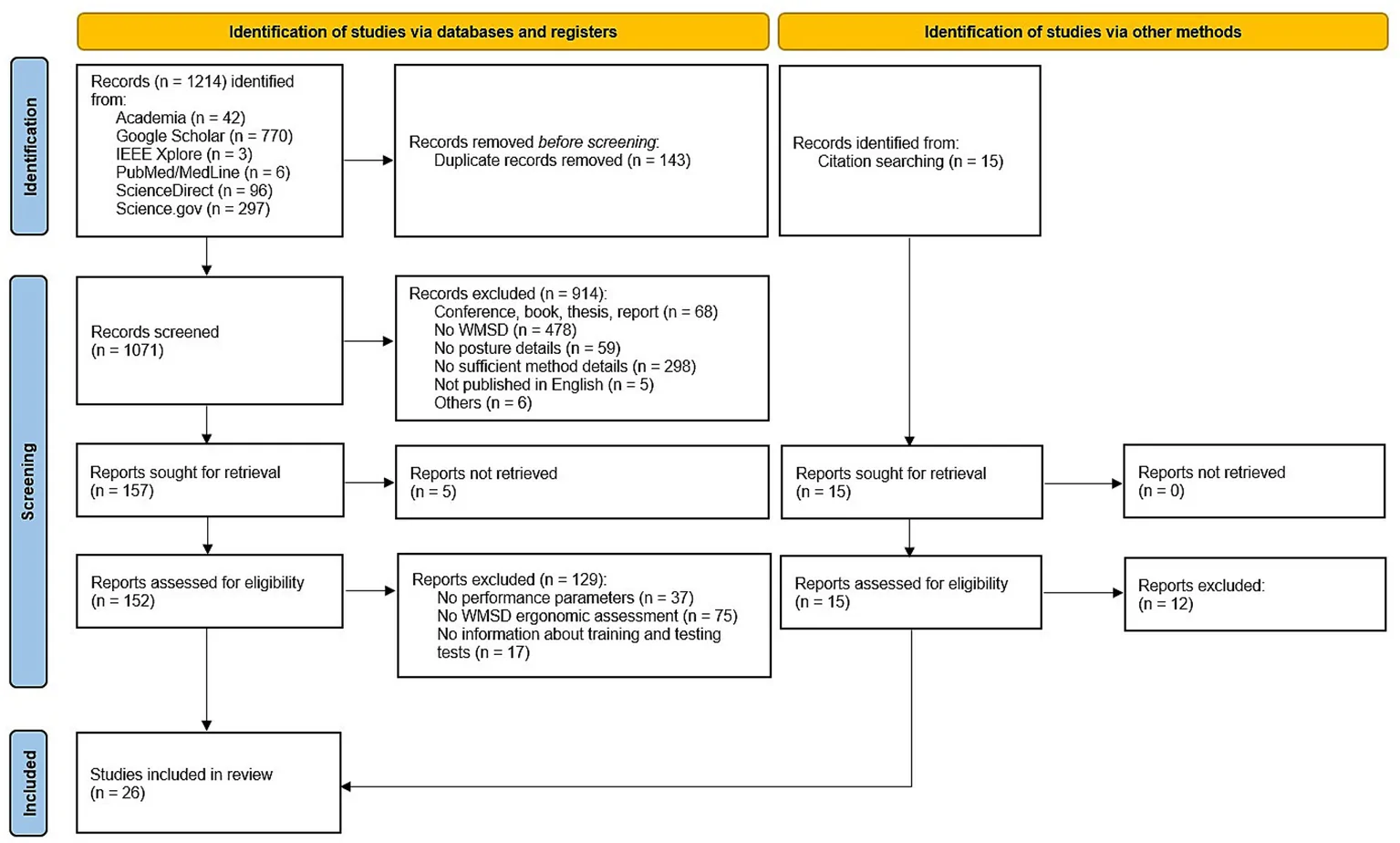
PRISMA flow diagram.

### 3.2 Study characteristics

Table 2 presents the characteristics of the 26 selected studies, including occupational activity, tasks performed and postures observed, the method for assessing WMSD risks, information regarding the sensors used and the AI method developed, as well as the number of subjects tested.

**Table 2:** Characteristics of the included studies.

Authors	Field	Task	Posture	WMSD assessment	Data acquisition method	Sensors position	Subjects Tested *n* (M, F)	AI Method	Algorithms	Validation type
Abobak et al. (2019)	Manufacturing	Manual industrial tasks	Whole body postures	RULA	Camera	Ext	6 (6, 0)	DL	ResNet, FCIS	Ext val
Antwi-Afari et al. (2018)	Construction	Construction activities	OW, SQ, STO, SS, OLK	APR	IP	Feet	10 (10, 0)	ML	ANN, DT, KNN, SVM	10-fold CV
Antwi-Afari et al. (2020)	Construction	HOL, CAR, LIF, LOW, PUS, PUL	ST	OSHA	IP, Acc	Feet	2 (2, 0)	ML	ANN, DT, KNN, RF, SVM	10-fold CV
Antwi-Afari et al. (2022)	Construction	REB	OW, SQ, STO, SS, OLK	APR	IP, Acc, Gyro	Feet	10 (10, 0)	DL	LSTM, Bi-LSTM, GRU	10-fold CV
Conforti et al. (2020)	Manufacturing	LIF, REL	Whole body postures	S vs. U	IMU	Sternum, pelvis, LL	26	ML	SVM	LOOCV
Cruciata et al. (2025)	Manufacturing	HAN, ASS	Whole body postures	RULA	Camera, IMU	Full-body	NA	DL	ViT	5-fold CV
Darvishi et al. (2017)	Industry	LIF, LOW, PUS, PUL	BT, BTW	S vs. U	Q, REBA, OME	No sensors	160 (92 LBP, 68\u00B0C)	ML	ANN, MLP, LR, KNN	Train-test split (80/20)
Davoudi Kakhki et al. (2025)	Manufacturing	LIF	ST, OW, BT	NIOSH	EMG	Upper-body	25 (15, 10)	DL	CNN, MLP, LSTM	Train-test split (80/20)
Donisi et al. (2021)	Manufacturing	LIF	ST, BT, SQ, OW	NIOSH	IMU	Lower back	7	ML	DT, RF, GB, AdaBoost, KNN, NB, MLP, SVM, LR	10-fold CV
Han et al. (2024)	Healthcare	PT	Whole body postures	REBA	Camera, IMU	Full-body	NA	DL	ST-GCN	Camera vs. IMU
Hossain et al. (2023)	All	All	Whole body postures	REBA	Human3.6 M dataset	Full-body	11 (Human3.6 M dataset)	DL	DNN	Train-val-test split (70/15/15)
Huang et al. (2024)	Manufacturing	ASS, HAN, PAC	Whole body postures	REBA	Camera	Ext	26 (20, 6)	DL	CNN	Train-test split (90/10)
Jiao et al. (2024)	Manufacturing	LIF, CAR	Whole body postures	REBA	Camera	Ext	9	DL	CPN	Int val
Kapse et al. (2024)	Agriculture	WEE, TRI, TRANSP	BT	RULA/REBA	Camera	Ext	6	ML, DL	CNN, VGG, Multiple Net, DT, SVM, RF, ANN	3-fold CV
Li et al. (2020)	All	All	Whole body postures	RULA	Human3.6 M dataset	Ext	NA	DL	DNN	5-fold CV
Nath et al. (2018)	Construction	LIF, LOW, CAR, PUS, PUL	Whole body postures	OSHA	IMU	UA, waist	2	ML	SVM	LOSOCV
Prisco et al. (2024)	All	LIF	ST, BT, SQ	S vs. U	IMU	Sternum	15 (9, 6)	ML	LR, SVM, DT, GB, RF, KNN, MLP, PNN	LOSOCV
Rahman et al. (2025)	Sport	All Sports	Whole body postures	REBA	Camera	Full-body	NA	DL	VGG, Multiple Net, IV3, Xception, ViSK-GAT	Train-val-test split (70/10/20)
Senjaya et al. (2023)	Manufacturing	ASS	Whole body postures	RULA	Camera	Full body, hands/fingers	12	DL	DNN, Bi-LSTM, CNN, HBU, HyNet	Train-test split (80/20)
Seo and Lee (2021)	Construction	Construction activities	ST, BB, AR, KN	OWAS	Kinect	Ext	8 (8, 0)	ML	SVM	Independent testing
Villalobos and Mac Cawley (2022)	Industry	BON	Wrist/hand motion	RULA	IMU	Wrists	20	ML	DT, SVM, RF, ET	Train-test split (70/30)
Wang et al. (2021)	Construction	LIF, CAR, LOW, HAN	Whole body postures	REBA	Camera	Ext	15 (9, 6)	DL	CNN	Train-test split (70/30)
Xiahou et al. (2023)	Construction	REB, BRI, CLE, PLA	OW, BT, BTW, LF	APR	Camera, IP, IMU, EEG	Ext, Feet, Hands	9 (7, 2)	DL	MLP, RNN, LSTM	10-fold CV
Zhang et al. (2018)	Construction	Construction activities	BB, AR, KN	OWAS	Camera	Ext	NA	ML	BP-ANN, DT, SVM, KNN, EC	5-fold CV
Zhao and Obonyo (2020)	Construction	DRI, PAI, ASS	BT, KN, LB, OW, SQ, ST	OWAS	IMU	Head, chest, UA, LL	4 (4, 0)	ML, DL	CLN, CNN, LSTM, SVM	Train-test split (90/10)
Zhao and Obonyo (2021)	Construction	INST, PUL, CLE, PAI, PLA	BT, KN, LB, OW, SQ, ST	OWAS	IMU	Head, chest, UA, LL	9	DL	CLN	Train-test split (90/10)

*TASK*: ASS, Assembly; BON, Boning; BRI, Bricklaying; CAR, Carrying; CLE, Cleaning; DRI, Drilling; HAN, Handling; HOL, Holding; INST, Installation; LIF, Lifting; LOW, Lowering; PAC, Packaging; PAI, Painting; PLA, Plastering; PT, Patient Transfer; PUL, Pulling; PUS, Pushing; REB, Rebar; TRANSP, Plant transportation in outdoor nursery; TRI, Trimming; WEE, Weeding; *Posture*: AR, Arm-raising; BB, Back-bending; BT, Bending posture; BTW, Body twisting; KN, Kneeling posture; LB, Lateral bending posture; LF, Leaning forward with single-leg supported; OLK, One-legged kneeling; OW, Overhead working posture; SQ, Squatting posture; SS, Semi-squatting; ST, Standing posture; STO, Stooping; *WMSD assessment*: APR, Awkward Posture Recognition; NIOSH, National Institute for Occupational Safety and Health; OSHA, Occupational Safety and Health Administration; OWAS, Ovako Working Posture Analysing System; REBA, Rapid Entire Body Assessment; RULA, Rapid Upper Limb Assessment; S vs U, Safe vs Unsafe posture; WMSD, Work-related musculoskeletal disorders; *Data Acquisition Method*: Acc, Accelerometer; EEG, Electroencephalography; EMG, Electromyography; Gyro, Gyroscope; IMU, Inertial Measurement Unit; IP, Insole Pressure; OME, Occupational medicine examination; Q, Questionnaire; *Sensors position*: Ext, extern; LL, Lower limb; UA, Upper arm; *Subjects Tested*: C, Control subjects; F, Female; LBP, Low Back Pain; M, Male; NA, Not Available; *AI Method*: DL, Deep Learning; ML, Machine Learning; *Algorithms*: ANN, Artificial Neural Network; AdaBoost, Adaptive Boosting; BP-ANN, Back-Propagation Artificial Neural Network; CLN, Convolutional LSTM model; CNN, Convolutional Neural Network; CPN, Cascaded Pyramid Network; DNN, Deep Neural Networks; DT, Decision Tree; EC, Ensemble Classifiers; ET, Extremely randomized Trees; FCIS, Fully Convolutional Instance Segmentation network; GB, Gradient Boosted Tree; GRU, Gated Recurrent Unit; HBU, Hybrid network of Bi-LSTM and Unidirectional LSTM; IV3, InceptionV3; KNN, K-Nearest Neighbors; LR, Logistic Regression; LSTM, Long Short-Term Memory; MLP, MultiLayer Perceptron; NB, Naïve Bayes Classifier; PNN, Probabilistic Neural Network; RF, Random Forest; RNN, Recurrent Neural Network; ST-GCN, Spatial Temporal Graph Convolutional Networks; SVM, Support Vector Machine; VGG, Visual Geometry Group; ViT, Vision Transformer; *Validation type*: CV, Cross-Validation; Ext val, External validation; Int val, Internal validation; LOOCV, Leave-One-Out Cross-Validation; LOSOCV, Leave-One-Subject-Out Cross-Validation.

Six occupational activities were represented: agriculture (1 study; Kapse et al., 2024), construction (10 studies; Wang et al., 2021; Antwi-Afari et al., 2022; Antwi-Afari et al., 2018; Antwi-Afari et al., 2020; Nath et al., 2018; Seo and Lee, 2021; Xiahou et al., 2023; Zhang et al., 2018; Zhao and Obonyo, 2020; Zhao and Obonyo, 2021), healthcare (1 study; Han et al., 2024), industry (2 studies; Darvishi et al., 2017; Villalobos and Mac Cawley, 2022), manufacturing (8 studies; Abobak et al., 2019; Conforti et al., 2020; Cruciata et al., 2025; Davoudi Kakhki et al., 2025; Donisi et al., 2021; Huang et al., 2024; Jiao et al., 2024; Senjaya et al., 2023), and sports (1 study; Rahman et al., 2025). Three studies were designed to be applicable to all occupational activities (Prisco et al., 2024; Hossain et al., 2023; Li et al., 2020). They were grouped under the heading “All.”

Thirteen postures were analyzed across the 26 studies: arm-raising, back-bending, bending posture, body twisting, kneeling posture, lateral bending posture, leaning forward with single-leg support, one-legged kneeling, overhead working posture, squatting posture, semi-squatting, standing, and stooping. These postures were observed during 21 different occupational tasks: assembly, boning, bricklaying, carrying, cleaning, drilling, handling, holding, installation, lifting, lowering, packaging, painting, patient transfer, plant transportation in an outdoor nursery, plastering, pulling, pushing, rebar work, trimming, and weeding.

Regarding the AI methods developed, 10 studies used a ML approach (Prisco et al., 2024; Antwi-Afari et al., 2018; Antwi-Afari et al., 2020; Nath et al., 2018; Seo and Lee, 2021; Zhang et al., 2018; Villalobos and Mac Cawley, 2022; Conforti et al., 2020; Davoudi Kakhki et al., 2025; Donisi et al., 2021), while 14 employed a DL approach (Wang et al., 2021; Antwi-Afari et al., 2022; Xiahou et al., 2023; Zhao and Obonyo, 2021; Han et al., 2024; Abobak et al., 2019; Cruciata et al., 2025; Davoudi Kakhki et al., 2025; Huang et al., 2024; Jiao et al., 2024; Senjaya et al., 2023; Rahman et al., 2025; Hossain et al., 2023; Li et al., 2020). Two studies compared the two AI methods (Kapse et al., 2024; Zhao and Obonyo, 2020). A wide variety of algorithms were tested. The most commonly used algorithms in the DL approach were CNN and LSTM, while those most commonly used in ML were SVM, DT, KNN, and RF. Considering the validation procedure, the authors used either cross-validation (x-fold, leave-one-out, or leave-one-subject-out) (Prisco et al., 2024; Antwi-Afari et al., 2022; Kapse et al., 2024; Nath et al., 2018; Xiahou et al., 2023; Zhang et al., 2018; Conforti et al., 2020; Cruciata et al., 2025; Donisi et al., 2021; Li et al., 2020), a train-test split with (Rahman et al., 2025; Hossain et al., 2023) or without a validation set (Wang et al., 2021; Zhao and Obonyo, 2020; Zhao and Obonyo, 2021; Darvishi et al., 2017; Villalobos and Mac Cawley, 2022; Davoudi Kakhki et al., 2025; Huang et al., 2024; Senjaya et al., 2023), or an independent test (Seo and Lee, 2021; Han et al., 2024; Abobak et al., 2019; Jiao et al., 2024). Sample sizes ranged from 2 to 160 subjects during an experimental protocol. One study used a dataset (the Human 3.6 M dataset) (Hossain et al., 2023), and 5 studies did not provide information about the sample (Zhang et al., 2018; Han et al., 2024; Cruciata et al., 2025; Rahman et al., 2025; Li et al., 2020).

The sensors used for data collection were either wearable sensors for 11 studies (Prisco et al., 2024; Antwi-Afari et al., 2022; Antwi-Afari et al., 2018; Antwi-Afari et al., 2020; Nath et al., 2018; Zhao and Obonyo, 2020; Zhao and Obonyo, 2021; Villalobos and Mac Cawley, 2022; Conforti et al., 2020; Davoudi Kakhki et al., 2025; Donisi et al., 2021), non-wearable sensors in 11 studies (Wang et al., 2021; Kapse et al., 2024; Seo and Lee, 2021; Zhang et al., 2018; Abobak et al., 2019; Huang et al., 2024; Jiao et al., 2024; Senjaya et al., 2023; Rahman et al., 2025; Hossain et al., 2023; Li et al., 2020), or hybrid solutions in 3 studies (Xiahou et al., 2023; Han et al., 2024; Cruciata et al., 2025). Among the wearable sensors, inertial measurement units (IMUs) with accelerometer, gyroscope and magnetometer and foot insole pressure sensors were primarily used. For non-wearable sensors, the authors overwhelmingly used cameras. Only the study by Darvishi et al. (2017) used a questionnaire coupled with a clinical examination rather than sensors.

Table 3 presents the performance parameters reported in each study. Only 3 studies evaluated the 5 metrics (Prisco et al., 2024; Antwi-Afari et al., 2022; Conforti et al., 2020). Seven studies reported four parameters, omitting specificity (Wang et al., 2021; Kapse et al., 2024; Nath et al., 2018; Cruciata et al., 2025; Davoudi Kakhki et al., 2025; Hossain et al., 2023; Li et al., 2020). Eight studies assessed AI method with only a single performance metric: either accuracy (5 studies; Antwi-Afari et al., 2020; Zhang et al., 2018; Han et al., 2024; Darvishi et al., 2017; Abobak et al., 2019) or F1 score (3 studies; Zhao and Obonyo, 2020; Zhao and Obonyo, 2021; Rahman et al., 2025). The most commonly studied performance metrics were accuracy (21 out of 26 studies) and F1 score (18 out of 26 studies). Sensitivity and precision were reported in 15 and 14 studies, respectively. Specificity was by far the least studied metric, as it was used in only 4 studies.

**Table 3:** Performance parameters reported in each selected study.

Authors	Accuracy	Specificity	Sensitivity	Precision	F1 score
Abobak et al. (2019)	X				
Antwi-Afari et al. (2018)	X		X		
Antwi-Afari et al. (2020)	X				
Antwi-Afari et al. (2022)	X	X	X	X	X
Conforti et al. (2020)	X	X	X	X	X
Cruciata et al. (2025)	X		X	X	X
Darvishi et al. (2017)	X				
Davoudi Kakhki et al. (2025)	X		X	X	X
Donisi et al. (2021)	X	X	X		
Han et al. (2024)	X				
Hossain et al. (2023)	X		X	X	X
Huang et al. (2024)			X	X	X
Jiao et al. (2024)			X	X	X
Kapse et al. (2024)	X		X	X	X
Li et al. (2020)	X		X	X	X
Nath et al. (2018)	X		X	X	X
Prisco et al. (2024)	X	X	X	X	X
Rahman et al. (2025)	X				X
Senjaya et al. (2023)					X
Seo and Lee (2021)	X		X	X	
Villalobos and Mac Cawley (2022)	X			X	X
Wang et al. (2021)	X		X	X	X
Xiahou et al. (2023)	X				X
Zhang et al. (2018)	X				
Zhao and Obonyo (2020)					X
Zhao and Obonyo (2021)					X

X indicates that authors reported at least one value for the considered performance metric.

### 3.3 Risk of bias

The PROBAST analysis revealed that all included studies had a high risk of bias, primarily due to domain 4, “Analysis,” and, in some cases, domain 1, “Participants” (Figures 3A,B). Several critical issues were identified. For the “Analysis” domain, these included an imprecise separation of training and test data, raising concerns about the risk of data leakage; and potential overfitting leading to an optimistic estimate of performance. None of the included studies conducted external validation on an independent cohort, which consistently resulted in a high risk of bias for domain 4. For the “Participants” domain, the main critical issues concern excessively small sample sizes and the inclusion of novice, non-working subjects. This assessment effectively limits the generalizability of the results to real-world professional environments, which justifies the high risk of bias.

**Figure 3:**
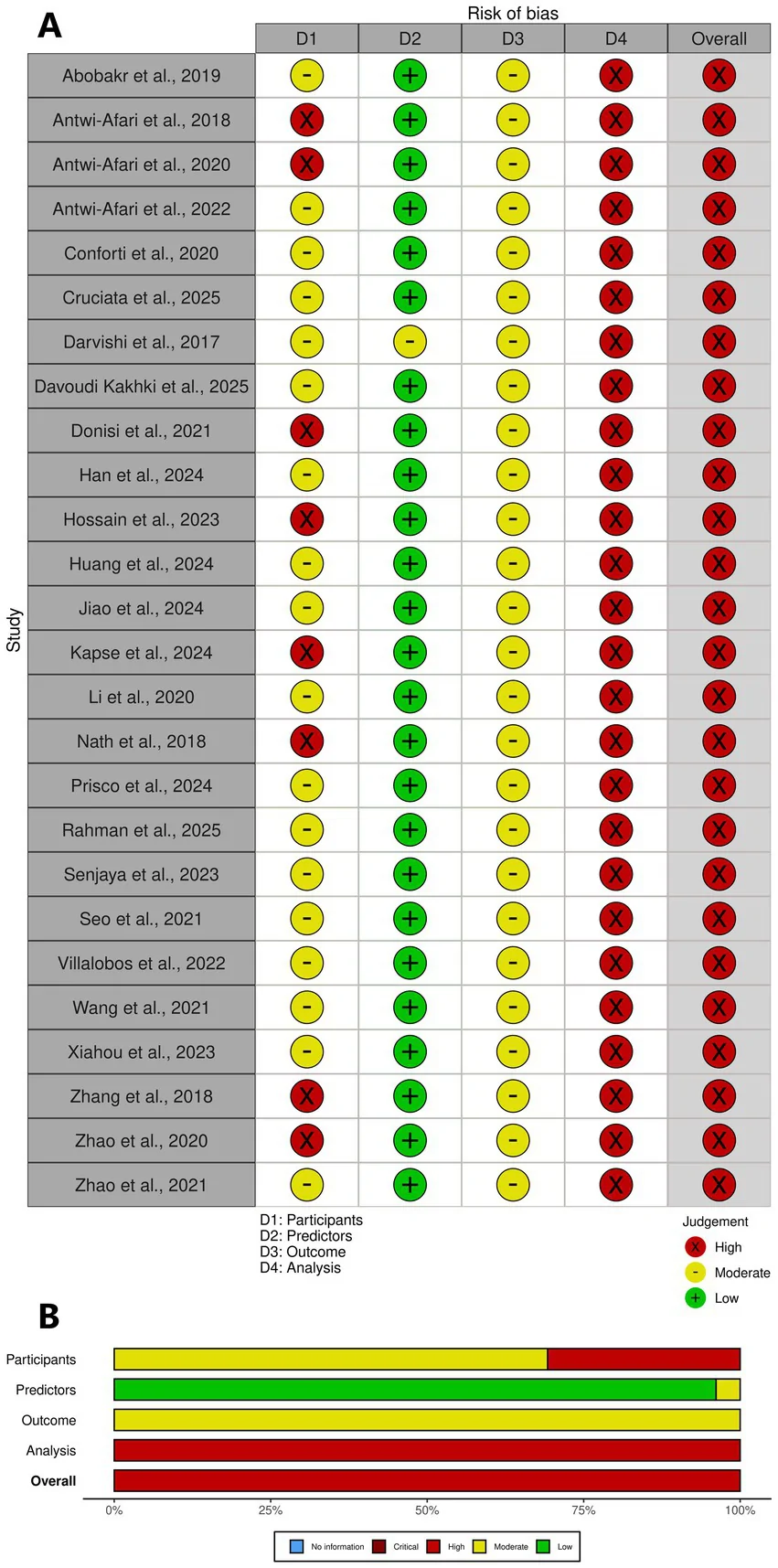
Results of risk of bias assessment. **(A)** Traffic-light plot. **(B)** Summary risk of bias assessment (Wang et al., 2021; Prisco et al., 2024; Antwi-Afari et al., 2022; Kapse et al., 2024; Antwi-Afari et al., 2018; Antwi-Afari et al., 2020; Nath et al., 2018; Seo and Lee, 2021; Xiahou et al., 2023; Zhang et al., 2018; Zhao and Obonyo, 2020; Zhao and Obonyo, 2021; Han et al., 2024; Darvishi et al., 2017; Villalobos and Mac Cawley, 2022; Abobak et al., 2019; Conforti et al., 2020; Cruciata et al., 2025; Davoudi Kakhki et al., 2025; Donisi et al., 2021; Huang et al., 2024; Jiao et al., 2024; Senjaya et al., 2023; Rahman et al., 2025; Hossain et al., 2023; Li et al., 2020).

### 3.4 Meta-analysis of performance parameters

The overall performance ranged from 85 to 95% (Table 4). Accuracy was the metric that presented the lowest values regardless of the transformation applied. The metric with the best pooled performance was precision for both the logit and Freeman-Tukey transformations, and the F1 score for the untransformed data. For all performance metrics, the highest values were obtained with the logit transformation (values ranging from 89.54 to 94.09%), and the lowest values were observed for the untransformed data (values ranging from 84.50 to 87.80%). The Freeman-Tukey transformation yielded intermediate values ranging from 86.87 to 90.47%. However, the choice of variance-stabilizing transformation had little influence on between-study heterogeneity. Regardless of the transformation applied, heterogeneity remained extremely high (I^2^ ≈ 100%), suggesting that the observed heterogeneity mainly reflects genuine differences between studies rather than statistical artefacts related to the scale of analysis (see Supplementary Figures S1–S5 for logit forest plots results).

**Table 4:** Comparison of pooled values and heterogeneity parameters obtained with each transformation for the five performance parameters.

Parameter	Transformation	Pooled	95% CI	τ^2^	I^2^	*n*
Accuracy	Untransformed	84.5%	82.6–86.3%	0.021	100.0%	241
	Logit	89.5%	87.8–91.1%	1.707	100.0%	
	Freeman-Tukey	86.9%	85.1–88.6%	0.04	100.0%	
Specificity	Untransformed	85.2%	81.5–88.8%	0.032	100.0%	98
	Logit	92.9%	89.6–95.2%	3.993	99.9%	
	Freeman-Tukey	88.3%	84.7–91.4%	0.064	100.0%	
Sensitivity	Untransformed	86.3%	83.3–89.2%	0.037	100.0%	167
	Logit	93.1%	90.8–94.8%	3.321	99.9%	
	Freeman-Tukey	89.6%	86.9–92.1%	0.073	100.0%	
Precision	Untransformed	87.2%	84.3–90.1%	0.029	100.0%	139
	Logit	94.1%	91.9–95.7%	3.437	99.9%	
	Freeman-Tukey	90.5%	87.9–92.8%	0.061	100.0%	
F1 score	Untransformed	87.8%	85.7–89.9%	0.015	100.0%	148
	Logit	92.4%	90.5–93.9%	1.874	99.9%	
	Freeman-Tukey	90.1%	88.1–91.9%	0.036	99.9%	

95% CI, 95% confidence interval; *n*, number of available data.

### 3.5 Transformation effect analysis

Table 5 summarizes the results of the statistical normality indicators for the untransformed values and the two transformations. The results showed that for the QQ correlation, the Shapiro–Wilk W statistic, skewness, and kurtosis, the logit transformation was the one that most closely approximated a normal distribution for all performance metrics, although the normality test was consistently significant. Furthermore, the logit transformation also reduced outliers from poorly fitted or potentially influential studies. Indeed, it produced no extreme residuals for accuracy and F1 score, unlike the untransformed values (5 for both metrics) or those with the Freeman-Tukey transformation (2 and 1, respectively, for accuracy and F1 score). These results suggest that the logit transformation provided the best overall model fit. However, it is interesting to note that no transformation had a significant impact on heterogeneity.

**Table 5:** Statistical comparison of three proportion transformations (UT, logit, and Freeman–Tukey) used to select the most appropriate transformation for the meta-analysis.

Performance metric	Statistical criterion	UT	Logit	FT	Preferred transformation
Accuracy	QQ correlation	0.931	0.993	0.983	Logit
	Shapiro–Wilk W	0.867	0.986	0.965	Logit
	Shapiro–Wilk p	<0.05	<0.05	<0.05	Equivalent
	Skewness	−1.403	0.325	−0.642	Logit
	Kurtosis	2	−0.32	0.118	FT
	Extreme Studentized residuals	5	0	2	Logit
	I^2^ (%)	99.999	99.998	99.997	Equivalent
Specificity	QQ correlation	0.84	0.973	0.92	Logit
	Shapiro–Wilk W	0.71	0.948	0.85	Logit
	Shapiro–Wilk p	<0.05	<0.05	<0.05	Equivalent
	Skewness	−2.74	0.066	−1.766	Logit
	Kurtosis	10.552	1.247	5.431	Logit
	Extreme Studentized residuals	2	2	2	Equivalent
	I^2^ (%)	100	99.983	99.998	Equivalent
Sensitivity	QQ correlation	0.809	0.973	0.899	Logit
	Shapiro–Wilk W	0.655	0.946	0.807	Logit
	Shapiro–Wilk p	<0.05	<0.05	<0.05	Equivalent
	Skewness	−2.911	−0.69	−2.041	Logit
	Kurtosis	10.13	1.478	5.85	Logit
	Extreme Studentized residuals	5	5	5	Equivalent
	I^2^ (%)	99.999	99.994	99.997	Equivalent
Precision	QQ correlation	0.837	0.983	0.926	Logit
	Shapiro–Wilk W	0.704	0.966	0.859	Logit
	Shapiro–Wilk p	<0.05	<0.05	<0.05	Equivalent
	Skewness	−2.705	−0.252	−1.644	Logit
	Kurtosis	10.557	0.367	4.659	Logit
	Extreme Studentized residuals	2	2	2	Equivalent
	I^2^ (%)	100	99.995	99.997	Equivalent
F1 score	QQ correlation	0.905	0.986	0.98	Logit
	Shapiro–Wilk W	0.82	0.97	0.959	Logit
	Shapiro–Wilk p	<0.05	<0.05	<0.05	Equivalent
	Skewness	−1.78	0.433	−0.758	Logit
	Kurtosis	3.483	−0.205	0.604	Logit
	Extreme Studentized residuals	5	0	1	Logit
	I^2^ (%)	99.999	99.993	99.995	Equivalent

DL, deep learning; ML, machine learning; FT, Freeman-Tukey; UT, untransformed.

### 3.6 Exploration of possible causes of heterogeneity

#### 3.6.1 Subgroup analysis – DL vs. ML

Overall performance ranged from 85 to 99% for DL and 77 to 87% for ML (Table 6). For accuracy, the performance of the two AI methods was comparable for both untransformed data and data transformed using the Freeman-Tukey transformation. Accuracy values appeared higher for the DL approach with the logit transformation (89.33% vs. 76.89%). However, no significant differences were observed irrespective of the transformation (*p* > 0.05). The meta-regression revealed that DL significantly outperformed ML for specificity, sensitivity, and precision, regardless of the transformation applied (all *p* ≤ 0.010), with differences ranging from 4 to 16%. The largest differences were observed for specificity with the untransformed data (16.30%) and the Freeman-Tukey transformation (14.48%). Regarding the F1 score, the difference reached the threshold for statistical significance only after the logit transformation (difference of 10.39%, *p* = 0.049), whereas Untransformed and Freeman–Tukey analyses remained non-significant. With the exception of accuracy using the DL approach, the Freeman-Tukey transformation yielded the highest performance for all metrics, while the lowest results were obtained with the logit transformation. Except for F1, the analysis demonstrated good robustness of the results, as similar effects were found whatever the transformation.

**Table 6:** AI method (DL vs. ML) comparison of pooled values and heterogeneity parameters obtained with each transformation for the five performance parameters.

Parameter	Transformation	AI method	Pooled	95% CI	τ^2^	I^2^	*n*	z	*p*(Wald)
Accuracy	Untransformed	DL	84.70%	81.50–87.90%	0.021	100.00%	87	0.200	0.841
	Untransformed	ML	84.40%	82.10–86.60%	0.02	100.00%	154		
	Logit	DL	89.33%	87.08–91.23%	1.847	99.98%	87	0.306	0.759
	Logit	ML	76.89%	76.12–77.64%	1.629	100.00%	154		
	Freeman-Tukey	DL	87.00%	83.98–89.76%	0.04	99.99%	87	0.121	0.903
	Freeman-Tukey	ML	86.80%	84.49–88.89%	0.04	100.00%	154		
Specificity	Untransformed	DL	98.90%	98.10–99.80%	0	100.00%	15	3.395	<0.001*
	Untransformed	ML	82.60%	78.50–86.70%	0.034	99.01%	83		
	Logit	DL	87.57%	83.41–90.80%	2.345	100.00%	15	9.133	<0.001*
	Logit	ML	81.59%	81.05–82.13%	1.83	96.58%	83		
	Freeman-Tukey	DL	99.33%	98.62–99.79%	0.004	99.99%	15	5.007	<0.001*
	Freeman-Tukey	ML	84.85%	80.70–88.58%	0.06	97.14%	83		
Sensitivity	Untransformed	DL	92.00%	89.00–95.10%	0.013	100.00%	65	2.969	0.003*
	Untransformed	ML	82.70%	78.40–87.00%	0.048	100.00%	102		
	Logit	DL	89.05%	84.70–92.28%	2.811	100.00%	65	4.491	<0.001*
	Logit	ML	79.20%	78.38–80.01%	2.998	99.11%	102		
	Freeman-Tukey	DL	94.63%	92.21–96.66%	0.039	100.00%	65	3.696	<0.001*
	Freeman-Tukey	ML	85.63%	81.25–89.45%	0.086	99.70%	102		
Precision	Untransformed	DL	91.20%	87.70–94.70%	0.019	100.00%	65	2.586	0.010*
	Untransformed	ML	83.60%	79.30–88.00%	0.035	100.00%	74		
	Logit	DL	89.05%	84.50–92.39%	3.649	100.00%	65	4.366	<0.001*
	Logit	ML	79.13%	78.21–80.01%	2.253	98.09%	74		
	Freeman-Tukey	DL	94.45%	91.67–96.66%	0.047	100.00%	65	3.512	<0.001*
	Freeman-Tukey	ML	86.05%	81.64–90.00%	0.065	98.54%	74		
F1 score	Untransformed	DL	88.70%	86.50–90.90%	0.013	100.00%	115	1.720	0.085
	Untransformed	ML	84.40%	79.10–89.60%	0.021	100.00%	33		
	Logit	DL	88.38%	82.56–92.44%	1.945	99.99%	115	1.968	0.049*
	Logit	ML	77.99%	77.40–78.57%	1.355	99.38%	33		
	Freeman-Tukey	DL	90.94%	88.89–92.79%	0.033	100.00%	115	1.754	0.079
	Freeman-Tukey	ML	86.73%	81.33–91.39%	0.043	99.27%	33		

*Indicates a significant difference. DL, deep learning; ML, machine learning; 95% CI, 95% confidence interval; *n*, number of available data.

#### 3.6.2 Subgroup analysis – sensor category

In this analysis, there was insufficient data for the specificity parameter to compare the different sensor categories. For the other four parameters, performance values ranged from 82 to 100% (Table 7). Regardless of the transformation used, the pooled values obtained with the logit transformation were higher than those computed with the Freeman-Tukey transformation. The untransformed values yielded the lowest pooled values. The paired mixed-effects meta-regression revealed relatively limited differences based on sensor type (see Supplementary Table S1 for the detailed comparative analysis). Overall accuracy was comparable among wearable, non-wearable, and hybrid devices; only the logit transformation showed a significant difference between wearable and non-wearable sensors (*p* = 0.020). In contrast, hybrid solutions consistently demonstrated higher sensitivity and precision than both wearable and non-wearable sensors (with pooled values exceeding 99%), with statistically significant differences observed for most transformations (*p* < 0.05). No significant differences were observed between wearable and non-wearable sensors for these two parameters. Regarding the F1 score, the results were less consistent. Only wearable systems systematically performed better than non-wearable systems for all three transformations (*p* < 0.05). A significant reduction in between-study heterogeneity was observed for the hybrid category for sensitivity and precision (I^2^ < 30% regardless of the transformation).

**Table 7:** Sensor category (wearable, non-wearable, hybrid) comparison of pooled values and heterogeneity parameters obtained with each transformation for the five performance parameters.

Parameter	Transformation	Sensors	Pooled	95% CI	τ^2^	I^2^	*n*
Accuracy	Untransformed	W	85.80%	83.50–88.20%	0.02	100.00%	142
	Untransformed	NW	84.10%	80.60–87.60%	0.008	99.83%	37
	Untransformed	H	82.10%	77.80–86.40%	0.028	99.91%	61
	Logit	W	90.92%	88.82–92.67%	1.752	100.00%	142
	Logit	NW	85.45%	81.58–88.63%	0.654	99.72%	37
	Logit	H	88.63%	84.06–92.00%	2.23	99.72%	61
	Freeman-Tukey	W	88.38%	86.12–90.47%	0.04	100.00%	142
	Freeman-Tukey	NW	84.42%	80.70–87.80%	0.018	99.75%	37
	Freeman-Tukey	H	84.92%	80.62–88.77%	0.049	99.63%	61
Sensitivity	Untransformed	W	84.80%	80.80–88.90%	0.046	100.00%	111
	Untransformed	NW	87.70%	83.40–91.90%	0.019	100.00%	48
	Untransformed	H	99.60%	99.40–99.80%	0	30.37%	8
	Logit	W	91.33%	87.96–93.83%	3.046	100.00%	111
	Logit	NW	94.24%	89.44–96.35%	3.005	99.95%	48
	Logit	H	99.39%	99.04–99.61%	0.022	7.77%	8
	Freeman-Tukey	W	87.93%	84.13–91.28%	0.083	100.00%	111
	Freeman-Tukey	NW	90.76%	86.53–94.27%	0.05	99.96%	48
	Freeman-Tukey	H	99.47%	99.18–99.70%	0	32.67%	8
Precision	Untransformed	W	86.70%	82.70–90.70%	0.032	100.00%	83
	Untransformed	NW	85.90%	81.20–90.60%	0.025	100.00%	48
	Untransformed	H	99.60%	99.40–99.70%	0	0.48%	8
	Logit	W	93.21%	90.04–95.42%	3.178	100.00%	83
	Logit	NW	92.93%	87.97–95.93%	3.195	99.98%	48
	Logit	H	99.46%	99.20–99.64%	0.063	18.79%	8
	Freeman-Tukey	W	89.63%	85.91–92.79%	0.064	100.00%	83
	Freeman-Tukey	NW	89.45%	84.78–93.40%	0.057	99.97%	48
	Freeman-Tukey	H	99.49%	99.27–99.67%	0	0.00%	8
F1 score	Untransformed	W	92.50%	90.50–94.50%	0.004	100.00%	43
	Untransformed	NW	85.40%	82.30–88.60%	0.018	100.00%	76
	Untransformed	H	86.70%	80.90–92.40%	0.023	99.94%	29
	Logit	W	94.56%	92.53–96.06%	1.046	100.00%	43
	Logit	NW	89.62%	86.80–92.27%	1.524	99.98%	76
	Logit	H	94.07%	88.52–97.03%	3.424	99.83%	29
	Freeman-Tukey	W	93.70%	91.67–95.46%	0.016	100.00%	43
	Freeman-Tukey	NW	87.60%	84.49–90.47%	0.038	99.98%	76
	Freeman-Tukey	H	90.12%	84.42–94.59%	0.051	99.69%	29

The analysis for specificity could not be conducted due to a lack of data. W, wearable sensors; NW, non-wearable sensors; H, hybrid solution; 95% CI, 95% confidence interval; *n*, number of available data.

#### 3.6.3 Subgroup analysis – occupational activity

Given the limited amount of available data, the analysis could not include data from the healthcare, agriculture, and sports sectors. For the other occupational activities—i.e., construction, industry, manufacturing, and “All”—the values for all performance metrics ranged from 82 to 100% (Table 8). The values obtained using the logit transformation were again higher for all parameters and all occupational activities, with the exception of “Industry,” where performance—as measured by the untransformed values of accuracy, sensitivity, precision, and F1 score—was highest (Figure 4). Significant inter-study heterogeneity persisted for all occupational activities, with the exception of accuracy for industry and “All” for which I^2^ was less than 50%.

**Table 8:** Occupational activity comparison of pooled values and heterogeneity parameters obtained with each transformation for the five performance parameters.

Parameter	Transformation	Occupational activity	Pooled	95% CI	τ^2^	I^2^	*n*
Accuracy	Untransformed	All	89.10%	88.00–90.20%	0.000	0.00%	2
	Untransformed	Construction	81.40%	77.60–85.30%	0.032	100.00%	85
	Untransformed	Industry	100.00%	100.00–100.00%	0.000	0.00%	8
	Untransformed	Manufacturing	86.70%	84.60–88.80%	0.013	99.99%	124
	Logit	All	88.21%	37.22–98.95%	0.047	31.49%	2
	Logit	Construction	87.63%	84.04–90.50%	1.881	100.00%	85
	Logit	Industry	97.27%	93.94–98.79%	0.337	55.54%	8
	Logit	Manufacturing	91.13%	88.95–92.91%	1.674	99.93%	124
	Freeman-Tukey	All	87.14%	25.91–91.14%	0.004	48.83%	2
	Freeman-Tukey	Construction	84.27%	80.55–87.67%	0.052	100.00%	85
	Freeman-Tukey	Industry	98.11%	95.58–99.61%	0.007	82.19%	8
	Freeman-Tukey	Manufacturing	88.70%	86.60–90.65%	0.032	99.97%	124
Specificity	Untransformed	All	-	-	-	-	-
	Untransformed	Construction	98.90%	98.10–99.80%	0.000	100.00%	15
	Untransformed	Industry	-	-	-	-	-
	Untransformed	Manufacturing	82.60%	78.50–86.70%	0.034	99.01%	83
	Logit	All	-	-	-	-	-
	Logit	Construction	99.61%	99.10–99.83%	2.345	100.00%	15
	Logit	Industry	-	-	-	-	-
	Logit	Manufacturing	87.57%	83.41–90.80%	1.830	96.58%	83
	Freeman-Tukey	All	-	-	-	-	-
	Freeman-Tukey	Construction	99.33%	98.62–99.79%	0.004	99.99%	15
	Freeman-Tukey	Industry	-	-	-	-	-
	Freeman-Tukey	Manufacturing	84.85%	80.70–88.58%	0.060	97.14%	83
Sensitivity	Untransformed	All	89.70%	84.40–95.00%	0.015	100.00%	24
	Untransformed	Construction	94.60%	91.60–97.70%	0.005	100.00%	23
	Untransformed	Industry	100.00%	100.00–100.00%	0.000	0.00%	8
	Untransformed	Manufacturing	84.10%	79.70–88.60%	0.050	100.00%	100
	Logit	All	94.22%	89.13–97.01%	2.027	99.96%	24
	Logit	Construction	96.70%	94.42–98.07%	1.317	100.00%	23
	Logit	Industry	98.64%	95.51–99.60%	1.380	77.78%	8
	Logit	Manufacturing	91.88%	87.98–94.59%	4.049	99.96%	100
	Freeman-Tukey	All	92.32%	87.34–96.14%	0.038	99.97%	24
	Freeman-Tukey	Construction	96.06%	93.50–98.00%	0.018	100.00%	23
	Freeman-Tukey	Industry	98.93%	96.73–99.93%	0.007	83.52%	8
	Freeman-Tukey	Manufacturing	87.54%	83.24–91.28%	0.092	99.98%	100
Precision	Untransformed	All	85.80%	78.70–92.80%	0.028	100.00%	24
	Untransformed	Construction	96.30%	94.60–98.10%	0.001	100.00%	22
	Untransformed	Industry	100.00%	100.00–100.00%	0.000	0.00%	8
	Untransformed	Manufacturing	85.80%	81.30–90.30%	0.036	100.00%	73
	Logit	All	92.19%	84.57–96.21%	2.539	99.99%	24
	Logit	Construction	98.17%	96.40–99.08%	2.237	100.00%	22
	Logit	Industry	97.98%	94.07–99.33%	1.155	80.76%	8
	Logit	Manufacturing	93.35%	89.60–95.81%	3.758	99.97%	73
	Freeman-Tukey	All	89.27%	82.33–94.59%	0.055	99.98%	24
	Freeman-Tukey	Construction	97.37%	95.58–98.69%	0.012	100.00%	22
	Freeman-Tukey	Industry	98.09%	94.94–99.74%	0.010	87.21%	8
	Freeman-Tukey	Manufacturing	89.08%	84.78–92.79%	0.073	99.98%	73
F1 score	Untransformed	All	86.90%	81.00–92.70%	0.019	100.00%	24
	Untransformed	Construction	87.70%	84.20–91.20%	0.015	100.00%	50
	Untransformed	Industry	100.00%	100.00–100.00%	0.000	0.00%	8
	Untransformed	Manufacturing	89.90%	87.10–92.60%	0.010	100.00%	53
	Logit	All	91.40%	85.20–95.15%	1.599	99.98%	24
	Logit	Construction	92.09%	88.90–94.42%	1.622	100.00%	50
	Logit	Industry	97.46%	93.31–99.06%	0.748	75.13%	8
	Logit	Manufacturing	94.08%	91.20–96.06%	2.052	99.99%	53
	Freeman-Tukey	All	89.51%	83.76–94.13%	0.040	99.97%	24
	Freeman-Tukey	Construction	89.88%	86.60–92.74%	0.032	100.00%	50
	Freeman-Tukey	Industry	97.92%	94.77–99.65%	0.009	86.72%	8
	Freeman-Tukey	Manufacturing	91.94%	89.27–94.22%	0.027	99.99%	53

95% CI, 95% confidence interval; *n*, number of available data.

**Figure 4:**
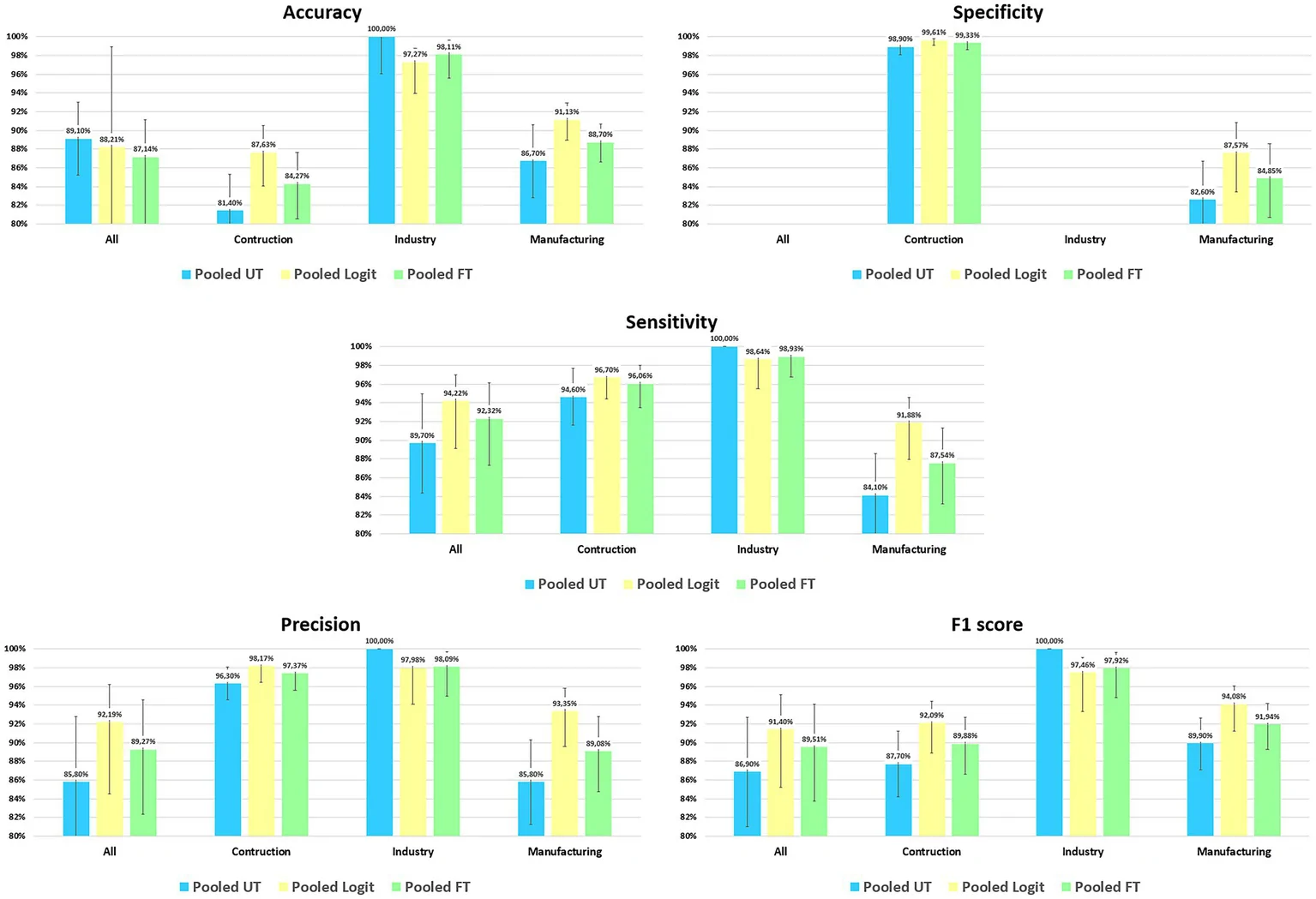
Pooled performance results for each of the five metrics, by identified professional activity, obtained for each transformation. The vertical bars represent the 95% confidence intervals. UT, Untransformed pooled value; Logit, Logit-transformed pooled value; FT, Freeman-Tukey-transformed pooled value.

Pairwise mixed-effects meta-regression showed that occupational activity had a limited influence on the overall performance of posture recognition systems (see Supplementary Table S2 for the detailed comparative analysis). The industrial sector generally showed the best performance estimates (values ranging from 97 to 100%), while the construction sector achieved comparable results in terms of sensitivity and precision. The manufacturing sector tended to achieve results slightly lower than those of industry and construction, although these differences were not consistently significant across the transformations.

#### 3.6.4 Subgroup analysis – ergonomic assessment tool

The values for all performance parameters ranged from 78 to 100% (Table 9; Figure 5). The values obtained using the logit transformation were higher for all parameters and all ergonomic assessment methods. Despite the subgroups, inter-study heterogeneity remained very high (I^2^ > 90%). A pairwise mixed-effects meta-regression revealed significant differences in performance depending on the ergonomic assessment tool used. However, no assessment tool consistently outperformed all others across all five performance parameters. In terms of accuracy, the RULA method performed significantly better than APR, NIOSH, OSHA, and REBA for all three transformations (values close to 95%, *p* < 0.05), suggesting a better overall ability to identify risky postures. With regard to sensitivity, the APR, REBA, and RULA methods all significantly outperformed NIOSH and the S vs. U classification, regardless of the transformation applied, thereby highlighting their superior ability to correctly detect postures that are genuinely at risk. In terms of specificity, the APR consistently outperformed (with a performance rate exceeding 98%, *p* < 0.05) both the NIOSH classification and the “Safe/Unsafe” (S vs. U) classification for all movements, indicating a greater ability to correctly identify postures that are not at risk (true negatives). Precision analyses showed that the S vs. U classification consistently yielded significantly lower values than those of the APR, REBA, and RULA for all three transformations, while few differences were observed among the other ergonomic tools. Finally, the OWAS method showed significantly lower F1 scores than several other ergonomic assessment methods, notably NIOSH, OSHA, and REBA, while REBA consistently outperformed OWAS.

**Table 9:** WMSD ergonomic assessment tool comparison of pooled values and heterogeneity parameters obtained with each transformation for the five performance parameters.

Parameter	Transformation	WMSD ergonomic assessment tool	Pooled	95% CI	τ^2^	I^2^	*n*
Accuracy	Untransformed	APR	82.20%	76.50–87.90%	0.033	100.00%	42
	Untransformed	NIOSH	87.70%	84.10–91.20%	0.009	100.00%	30
	Untransformed	OSHA	79.90%	74.30–85.50%	0.031	100.00%	41
	Untransformed	OWAS	93.30%	34.90–100.00%	0.004	99.96%	2
	Untransformed	REBA	83.80%	79.80–87.80%	0.011	99.40%	30
	Untransformed	RULA	93.60%	90.70–96.50%	0.005	100.00%	27
	Untransformed	S vs. U	86.10%	82.60–89.50%	0.016	99.04%	57
	Logit	APR	89.07%	83.52–92.91%	2.309	99.97%	42
	Logit	NIOSH	90.43%	86.74–93.18%	0.937	99.97%	30
	Logit	OSHA	85.08%	79.74–89.20%	1.378	100.00%	41
	Logit	OWAS	95.03%	0.02–100.00%	1.585	99.90%	2
	Logit	REBA	85.61%	81.85–88.71%	0.514	98.93%	30
	Logit	RULA	96.75%	94.16–98.21%	2.139	99.79%	27
	Logit	S vs. U	91.02%	87.25–93.76%	1.869	97.51%	57
	Freeman-Tukey	APR	85.28%	79.75–90.12%	0.056	99.99%	42
	Freeman-Tukey	NIOSH	88.95%	85.35–92.05%	0.020	99.99%	30
	Freeman-Tukey	OSHA	82.41%	76.86–87.34%	0.048	100.00%	41
	Freeman-Tukey	OWAS	94.17%	0.53–100.00%	0.019	99.95%	2
	Freeman-Tukey	REBA	84.71%	80.86–88.19%	0.018	99.11%	30
	Freeman-Tukey	RULA	95.37%	92.53–97.56%	0.022	99.53%	27
	Freeman-Tukey	S vs. U	88.32%	84.71–58.08%	0.038	97.04%	57
Specificity	Untransformed	APR	98.90%	98.10–99.80%	0.000	100.00%	15
	Untransformed	NIOSH	86.60%	81.90–91.30%	0.013	97.90%	27
	Untransformed	OSHA					
	Untransformed	OWAS					
	Untransformed	REBA					
	Untransformed	RULA					
	Untransformed	S vs. U	80.60%	75.00–86.30%	0.044	99.12%	56
	Logit	APR	99.61%	99.10–99.83%	2.345	100.00%	15
	Logit	NIOSH	89.33%	84.83–92.61%	0.916	95.13%	27
	Logit	OSHA					
	Logit	OWAS					
	Logit	REBA					
	Logit	RULA					
	Logit	S vs. U	86.70%	80.12–91.35%	2.531	96.92%	56
	Freeman-Tukey	APR	99.33%	98.62–99.79%	0.004	99.99%	15
	Freeman-Tukey	NIOSH	88.00%	83.46–91.89%	0.026	95.59%	27
	Freeman-Tukey	OSHA					
	Freeman-Tukey	OWAS					
	Freeman-Tukey	REBA					
	Freeman-Tukey	RULA					
	Freeman-Tukey	S vs. U	83.16%	77.20–88.38%	0.076	97.09%	56
Sensitivity	Untransformed	APR	94.10%	89.90–98.30%	0.006	100.00%	16
	Untransformed	NIOSH	87.60%	84.10–91.10%	0.008	99.99%	30
	Untransformed	OSHA					
	Untransformed	OWAS					
	Untransformed	REBA	95.90%	93.50–98.30%	0.003	100.00%	20
	Untransformed	RULA	93.40%	89.10–97.80%	0.014	100.00%	31
	Untransformed	S vs. U	78.60%	71.30–86.00%	0.076	100.00%	56
	Logit	APR	96.52%	93.84–98.05%	1.258	100.00%	16
	Logit	NIOSH	89.96%	86.16–92.81%	0.872	99.94%	30
	Logit	OSHA					
	Logit	OWAS					
	Logit	REBA	97.62%	95.16–98.85%	1.716	99.96%	20
	Logit	RULA	97.98%	95.88–99.02%	3.485	98.75%	31
	Logit	S vs. U	86.70%	77.42–92.55%	4.904	97.56%	56
	Freeman-Tukey	APR	95.46%	92.11–97.95%	0.018	100.00%	16
	Freeman-Tukey	NIOSH	88.58%	84.99–91.78%	0.019	99.98%	30
	Freeman-Tukey	OSHA					
	Freeman-Tukey	OWAS					
	Freeman-Tukey	REBA	97.14%	94.86–98.76%	0.015	99.94%	20
	Freeman-Tukey	RULA	96.33%	93.10–98.57%	0.039	98.76%	31
	Freeman-Tukey	S vs. U	81.87%	73.76–88.77%	0.132	98.29%	56
Precision	Untransformed	APR	96.10%	94.00–98.30%	0.002	100.00%	15
	Untransformed	NIOSH	94.70%	83.10–100.00%	0.002	100.00%	3
	Untransformed	OSHA					
	Untransformed	OWAS					
	Untransformed	REBA	91.60%	84.40–98.90%	0.024	100.00%	20
	Untransformed	RULA	93.10%	88.90–97.30%	0.013	100.00%	31
	Untransformed	S vs. U	81.90%	76.40–87.40%	0.042	99.12%	56
	Logit	APR	98.21%	95.88–99.24%	2.405	100.00%	15
	Logit	NIOSH	96.29%	57.59–99.80%	1.41	100.00%	3
	Logit	OSHA					
	Logit	OWAS					
	Logit	REBA	97.41%	93.05–99.06%	4.016	99.99%	20
	Logit	RULA	97.58%	95.32–98.77%	3.088	98.66%	31
	Logit	S vs. U	87.72%	81.61–92.00%	2.456	96.45%	56
	Freeman-Tukey	APR	97.18%	94.86–98.82%	0.012	100.00%	15
	Freeman-Tukey	NIOSH	95.50%	77.70–99.59%	0.012	100.00%	3
	Freeman-Tukey	OSHA					
	Freeman-Tukey	OWAS					
	Freeman-Tukey	REBA	95.20%	89.63–98.73%	0.053	99.98%	20
	Freeman-Tukey	RULA	95.82%	92.53–98.19%	0.037	98.67%	31
	Freeman-Tukey	S vs. U	84.27%	78.53–89.27%	0.073	96.94%	56
F1 score	Untransformed	APR	87.20%	82.60–91.80%	0.019	100.00%	36
	Untransformed	NIOSH	94.40%	81.40–100.00%	0.003	100.00%	3
	Untransformed	OSHA					
	Untransformed	OWAS	82.80%	79.50–86.10%	0.002	99.56%	8
	Untransformed	REBA	92.90%	88.10–97.60%	0.011	100.00%	21
	Untransformed	RULA	88.80%	85.80–91.90%	0.013	100.00%	59
	Untransformed	S vs. U	92.60%	89.30–95.90%	0.001	86.42%	8
	Logit	APR	92.17%	88.16–94.90%	1.804	100.00%	36
	Logit	NIOSH	96.02%	57.69–99.77%	1.339	100.00%	3
	Logit	OSHA					
	Logit	OWAS	83.06%	79.87–85.84%	0.064	99.44%	8
	Logit	REBA	96.24%	92.47–98.16%	1.959	99.99%	21
	Logit	RULA	93.57%	90.52–95.69%	2.183	99.99%	59
	Logit	S vs. U	92.88%	89.19–95.38%	0.247	83.24%	8
	Freeman-Tukey	APR	89.63%	85.35–93.25%	0.037	100.00%	36
	Freeman-Tukey	NIOSH	95.20%	76.10–99.51%	0.014	100.00%	3
	Freeman-Tukey	OSHA					
	Freeman-Tukey	OWAS	82.94%	79.67–85.98%	0.002	99.50%	8
	Freeman-Tukey	REBA	94.94%	90.99–97.77%	0.029	99.97%	21
	Freeman-Tukey	RULA	91.28%	88.38–93.84%	0.035	99.97%	59
	Freeman-Tukey	S vs. U	92.64%	89.14–95.46%	0.004	84.15%	8

95% CI, 95% confidence interval; *n*, number of available data.

**Figure 5:**
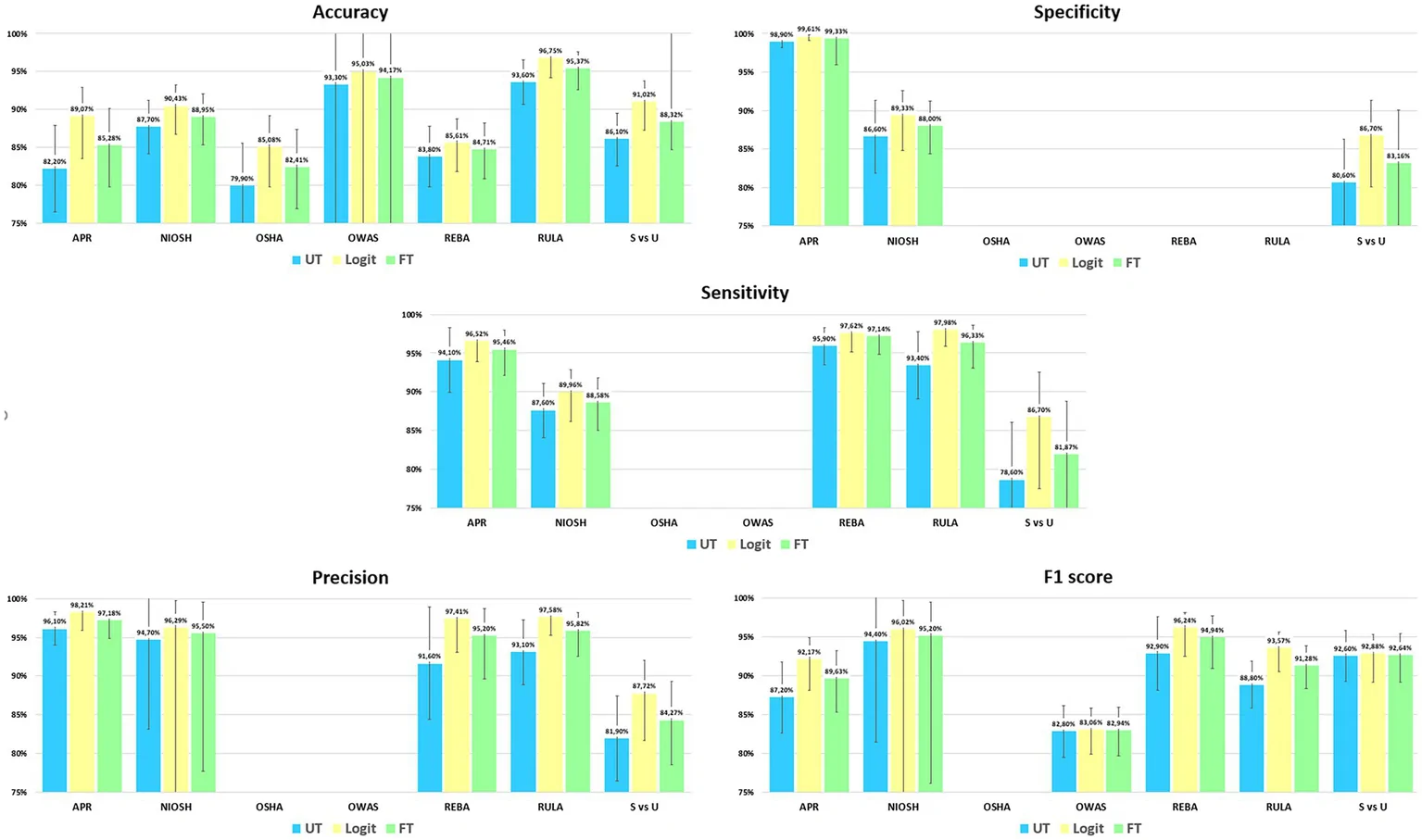
Pooled performance results for each of the five metrics, by ergonomic evaluation method of WMSD risk, obtained for each transformation. The vertical bars represent the 95% confidence intervals. UT, Untransformed pooled value; Logit, Logit-transformed pooled value; FT, Freeman-Tukey-transformed pooled value; APR, Awkward Posture Recognition; NIOSH, National Institute for Occupational Safety and Health; OSHA, Occupational Safety and Health Administration; OWAS, Ovako Working Posture Analysing System; REBA, Rapid Entire Body Assessment; RULA, Rapid Upper Limb Assessment; S vs. U, Safe vs. unsafe posture.

In summary, these results suggest that the RULA method offers the best classification accuracy, that REBA and RULA achieve the highest sensitivity, that APR has the highest specificity, that OWAS is associated with the lowest F1 score, and that S vs. U showed lower precision than APR, REBA, and RULA.

### 3.7 Sensibility analysis

Leave-one-out sensitivity analyses demonstrated excellent robustness of the results regardless of the performance parameter and transformation considered (Table 10). Excluding each study sequentially resulted in changes in the pooled values less than 1%. The largest variation was observed for specificity with UT, with a maximum difference of 1.14%. Similarly, heterogeneity remained essentially unchanged, with only negligible variations in τ^2^ and consistently high I^2^ values. These results indicate that no single study disproportionately influenced the overall results.

**Table 10:** Sensitivity analysis by leave-one-out – pooled performance, and heterogeneity for each metric and transformation.

Parameter	Comparison	Pooled performance			τ^2^			I^2^		
		UT	Logit	FT	UT	Logit	FT	UT	Logit	FT
Accuracy	Min	84.41%	89.36%	86.78%	0.019	1.644	0.038	100.0%	100.0%	100.0%
	Max	84.73%	89.64%	87.05%	0.021	1.712	0.040	100.0%	100.0%	100.0%
	Difference	0.31%	0.28%	0.27%	0.001	0.068	0.002	0.00%	0.00%	0.00%
Specificity	Min	85.03%	92.58%	88.03%	0.025	3.619	0.053	100.0%	99.99%	100.0%
	Max	86.17%	93.30%	88.97%	0.033	3.963	0.065	100.0%	100.0%	100.0%
	Difference	1.14%	0.72%	0.94%	0.008	0.344	0.012	0.00%	0.01%	0.00%
Sensitivity	Min	86.17%	93.03%	89.50%	0.033	2.969	0.066	100.0%	100.0%	100.0%
	Max	86.82%	93.34%	90.02%	0.037	3.206	0.074	100.0%	100.0%	100.0%
	Difference	0.64%	0.32%	0.53%	0.005	0.237	0.007	0.00%	0.00%	0.00%
Precision	Min	87.08%	93.94%	90.34%	0.023	3.138	0.053	100.0%	100.0%	100.0%
	Max	87.92%	94.30%	90.96%	0.029	3.404	0.062	100.0%	100.0%	100.0%
	Difference	0.84%	0.36%	0.62%	0.006	0.266	0.009	0.00%	0.00%	0.00%
F1 score	Min	87.71%	92.18%	89.96%	0.014	1.802	0.033	100.0%	100.0%	100.0%
	Max	88.14%	92.50%	90.34%	0.015	1.894	0.036	100.0%	100.0%	100.0%
	Difference	0.43%	0.32%	0.38%	0.002	0.093	0.003	0.00%	0.00%	0.00%

Min, minimum value; Max, maximum value; UT, Untransformed data; Logit, logit-transformed data; FT, Freeman-Tukey transformed data.

When considering all the available data, an imbalance was observed among the studies. Some of them proposed a single optimized solution, while others evaluated several. In the latter cases, the different solutions were evaluated using the same participants and the same dataset. They are therefore not statistically independent, which may introduce bias in the estimation of combined performance. To test this effect, a second sensitivity analysis was performed by retaining only the best-performing algorithm from each study (Table 11). Compared with the primary analysis including all reported algorithms, pooled performance increased substantially for accuracy (+7.8% to +11.1%), sensitivity (+5.3% to +11.1%), precision (+3.5% to +9.6%), and F1 score (+5.5% to +7.7%). These findings indicate that including multiple algorithms from the same study provides a more conservative estimate of model performance, whereas restricting the analysis to the best-performing algorithm yields substantially higher pooled estimates.

**Table 11:** Sensitivity analysis by considering only the best algorithm – pooled performance, and heterogeneity for each metric and transformation.

Parameter	Transformation	Pooled	95% CI	τ^2^	I^2^	*n*	Diff *
Accuracy	Untransformed	95.5%	92.9–98.0%	0.002	100%	18	11.00%
	Logit	97.3%	94.6–98.7%	2.010	99.98%		7.80%
	Freeman-Tukey	96.3%	93.5–98.4%	0.015	99.99%		9.40%
Specificity	Untransformed	97.9%	92.0–100.0%	0.001	96.32%	4	12.70%
	Logit	92.6%	0.4–100.0%	25.36	99.76%		−0.30%
	Freeman-Tukey	80.1%	0.4–100.0%	0.427	99.85%		−8.20%
Sensitivity	Untransformed	97.4%	95.5–99.2%	0.001	100%	16	11.10%
	Logit	98.4%	96.3–99.3%	1.869	99.97%		5.30%
	Freeman-Tukey	98.0%	96.1–99.3%	0.011	99.97%		8.40%
Precision	Untransformed	96.8%	94.3–99.2%	0.002	100%	14	9.60%
	Logit	97.6%	87.5–99.6%	8.176	99.99%		3.50%
	Freeman-Tukey	94.0%	80.6–99.9%	0.128	100%		3.50%
F1 score	Untransformed	95.5%	92.9–98.1%	0.002	100%	17	7.70%
	Logit	97.9%	94.7–99.2%	2.723	99.98%		5.50%
	Freeman-Tukey	96.9%	94.0–98.8%	0.018	99.98%		6.80%

^*^The difference was computed by subtracting the data obtained by considering all the algorithms in the included studies from the data obtained using only the best algorithm from each study. 95% CI, 95% confidence interval; *n*, number of available data.

### 3.8 Publication bias

The symmetry of the funnel plots was examined, and Egger’s test was performed for each performance metric and each transformation. Figure 6 shows the funnel plots for the logit transformation and the test results for all transformations. Twelve of the fifteen distributions showed asymmetry with a significant Egger’s test, indicating the presence of publication bias or effects related to small study sizes. Only Accuracy and F1 score with FT, and specificity with Logit, showed no asymmetry (Egger’s test *p* > 0.05). Funnel plots for the untransformed data and for the Freeman-Tukey transformation are presented in Supplementary Figures S6, S7.

**Figure 6:**
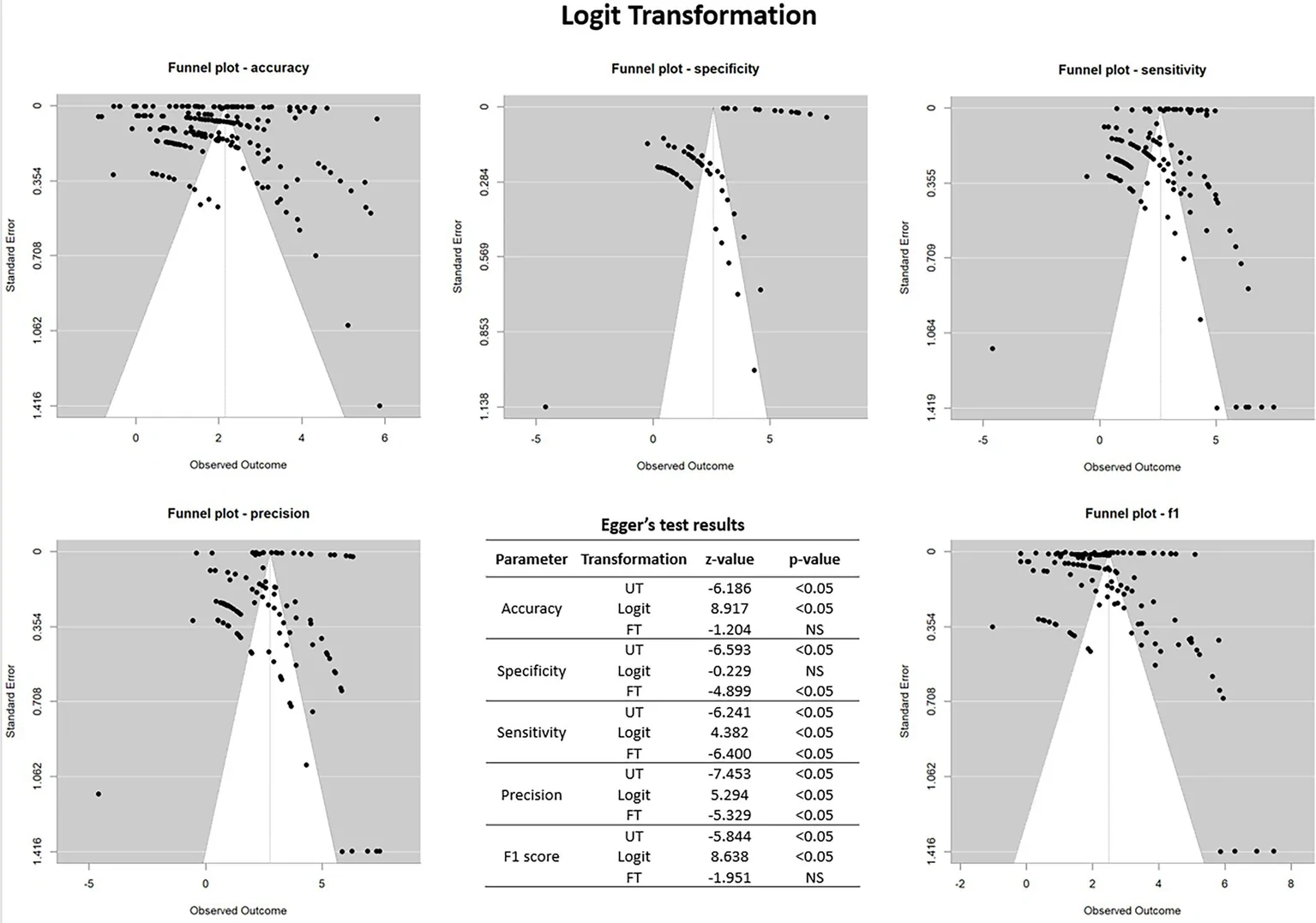
Funnel plots and corresponding Egger’s test results for each performance parameter with logit transformation.

### 3.9 Level of evidence

Accuracy studies of diagnostic tests initially provide a high level of evidence. However, among the five GRADE domains, three consistently presented a serious risk: indirectness, imprecision, and risk of bias, regardless of the performance measure or transformation used. These results can be explained by the fact that some studies included small samples of subjects—sometimes novices—or tested activities that were too far removed from real-world conditions. Furthermore, the PROBAST analysis revealed a high risk of bias for all included studies, and very high heterogeneity (I^2^ > 99%) was observed (reflecting very high inter-study variability). These results led to a very low overall level of evidence for all performance measures, regardless of the transformation method used. Table 12 reports the GRADE results for the logit transformation. The results for the other two transformations are provided in Supplementary Tables S3, S4.

**Table 12:** Detailed GRADE analysis for logit transformation.

Performance parameter	Number of studies	Certainty assessment						Effect			Overall level of evidence
		Study design	Publication bias (Egger’s Test)	Indirectness ^a^	Inconsistency ^b^	Imprecision ^c^	Risk of bias^d^	*n*	Event rate	(95% CI)	
Accuracy	21	DTAS	Serious (*p* < 0.001)	Serious	Serious (I^2^ = 100%)	Not serious	Serious	241	89.5%	87.8–91.1%	Very low ⬤◯◯◯
Specificity	4	DTAS	Not serious (*p* = 0.819)	Serious	Serious (I^2^ = 99.9%)	Not serious	Serious	98	92.9%	89.6–95.2%	Very low ⬤◯◯◯
Sensitivity	15	DTAS	Serious (*p* < 0.001)	Serious	Serious (I^2^ = 99.9%)	Not serious	Serious	167	93.1%	90.8–94.8%	Very low ⬤◯◯◯
Precision	14	DTAS	Serious (*p* < 0.001)	Serious	Serious (I^2^ = 99.9%)	Not serious	Serious	139	94.1%	91.9–95.7%	Very low ⬤◯◯◯
F1 score	18	DTAS	Serious (*p* < 0.001)	Serious	Serious (I^2^ = 99.9%)	Not serious	Serious	148	92.4%	90.5–93.9%	Very low ⬤◯◯◯

DTAS, Diagnostic test accuracy study; *n*, number of available data; 95% CI, 95% confidence interval; *n*, number of available data.

GRADE overall quality significance: ⬤◯◯◯ = Very low; ⬤⬤◯◯ = Low; ⬤⬤⬤◯ = Moderate; ⬤⬤⬤⬤ = High.

^a^Studied population correspond to the population in study.

^b^Serious if I^2^ > 50%.

^c^Serious if 95% CI Range Difference > 50% of Event Rate.

^d^Studies have at least a fair critical appraisal score.

## 4 Discussion

The objective of this meta-analysis was to provide an overview of the performance of AI-based methods for recognizing work postures, with the aim of preventing the occurrence of WMSDs in occupational activities. Twenty-six studies were included, covering six sectors—agriculture, construction, healthcare, industry, manufacturing, and sports—and encompassing numerous tasks and postures. Performance results were pooled and compared by AI method (ML vs. DL), by occupational activity, by sensor category, and by ergonomic assessment tools.

### 4.1 Performance results for WMSD prediction

The results showed that AI systems performed well in detecting WMSD risks based on posture analysis during various work activities. The combined values for each performance metric were fairly consistent and pooled close to 90%. Among the five performance metrics, sensitivity is of particular interest. It reflects the ability to correctly detect a risky posture. In this study, sensitivity reached 93.1% using logit transformation when considering all data and exceeded 97% when considering only the best solutions. This result is significant because it indicates that the systems are able to detect nearly all risky postures. Under these conditions, fewer than 10% of these postures went undetected. This error can have a significant impact that should not be underestimated when undetected postures are repeated or maintained over a long period. Consequently, a sensitivity close to 100% helps reduce the number of undetected high-risk postures. Maximizing this parameter is consistent with recommendations set forth, particularly in medical AI, to avoid the serious consequences of an undetected positive case (Parikh et al., 2008; Narteni et al., 2022). The second expected feature of a WMSD prevention system is to limit false alarms, which was observed with precision and specificity rates close to 90%. These results ensure a high level of trust in the system among users and prevent fatigue caused by repeated alarms. However, the complete elimination of false alarms is not a primary objective. Classifying a non-risky posture as a risky one will result in an overall overestimation of the risk level at the workstation during a delayed assessment or will trigger an unnecessary alert during real-time monitoring. In the first case, since the analysis is conducted over a long period, the slight increase caused by a few non-risky postures will not significantly alter the assessment of WMSD risk. In the second case, a real-time alert for a non-risky posture will help maintain a high level of attention among operators to prevent risky postures. It is mainly in this context that the false alarm rate must remain low to avoid a loss of trust (Kangas et al., 2014). The high F1 scores reflect the detection systems’ effective balance between sensitivity and precision and demonstrate the promising potential of AI approaches for ergonomic monitoring.

However, these results should be interpreted with caution given the very low level of confidence highlighted by the GRADE analysis. Significant heterogeneity and numerous methodological weaknesses were identified in the primary studies, particularly regarding the selection of novice participants, experimental conditions that were too far removed from real-world work conditions, and the lack of external validation under real-world conditions. These limitations restrict the generalizability of the results and necessitate further research to confirm the observed findings.

### 4.2 Effects of transformations

One of the main methodological findings of this study is the consistent superiority of the logit transformation over approaches using raw data or the Freeman-Tukey method. For all five performance indicators, the logit transformation consistently produced distributions that were closer to normality. However, the Shapiro–Wilk test (which tests for normality) was statistically significant, indicating that the distribution remained non-normal regardless of the transformation (*p* < 0.05). It has been shown that the Shapiro–Wilk test is the most powerful test but that it readily detects very slight deviations from normality when the sample size is large (Razali and Wah, 2011). Because it is easily significant, the Shapiro–Wilk test is often used in conjunction with other descriptive measures of normality (Ghasemi and Zahediasl, 2012). With a higher correlation on the Q-Q correlation (0.993 vs. 0.983 and 0.931), a higher Shapiro–Wilk W statistic, skewness values consistently closer to zero, and a lower number of extreme studentized residuals, the logit transformation yielded the best results for approximating a normal distribution.

However, it should be noted that the transformation method had no effect on inter-study heterogeneity, as the I^2^ index remained close to 100%. This finding indicates that the substantial heterogeneity observed in this meta-analysis reflects genuine methodological differences among the studies, rather than artifacts induced by the statistical transformation.

In conclusion, the logit transformation provides a better statistical representation of the performance of AI systems for estimation using a random-effects model. Thus, this approach could be used as the primary method in meta-analyses, as it is naturally better suited for analyzing proportions between 80 and 100%. The other two approaches could then be used as sensitivity analyses to test the robustness of the presented results.

### 4.3 Exploration of causes of heterogeneity – subgroup analyses

Significant heterogeneity was observed among the five performance parameters. Subgroup analyses were conducted to explore various potential sources that might explain the differences observed across studies (Cochrane Training, 2021; Thompson, 1994) through the following comparisons: AI method, sensor type, occupational activities, and ergonomic tools.

#### 4.3.1 AI methods comparison

The first analysis was conducted to compare the two AI methods, DL and ML. Regardless of the transformation, the meta-regression revealed that DL significantly outperformed ML on three performance metrics—specifically, specificity, sensitivity, and precision—with differences ranging from 4 to 16% (*p* < 0.05). Thus, these approaches appear to be more effective for preventing WMSDs in occupational activities. This result is consistent with previous studies indicating superior performance of DL methods (LeCun et al., 2015; Abrol et al., 2021; Gorce and Jacquier-Bret, 2026b). It could be explained by these approaches’ greater ability to automatically process large amounts of complex data (LeCun et al., 2015). This is particularly true for posture analyses, which require multiple spatial data points contained in images or extracted from sensors to reconstruct the human skeleton (involving numerous segments and degrees of freedom) (Krizhevsky et al., 2017). However, this comparison introduces a bias related to the datasets. Indeed, in the comparison, the studies used different datasets to train their respective models (ML and DL). Yet, the dataset influences model performance (Althnian et al., 2021). Future studies are therefore necessary to refine the observed differences by comparing the two methods using common training, validation, and test datasets.

#### 4.3.2 Sensor categories

The meta-regression showed that hybrid solutions outperformed both wearable and non-wearable devices in terms of sensitivity and accuracy (pooled values > 99%) for most transformations (*p* < 0.05). This result can be explained by the fact that hybrid solutions combine both kinematic information provided by IMUs and spatial information provided by vision-based systems (e.g., cameras). Their combination offers richer information that improves the performance of AI models through multimodal fusion (Seng et al., 2023). Information redundancy also helps compensate for errors or missing data, thereby increasing the accuracy, robustness, and stability of the results (Majumder and Kehtarnavaz, 2021), as evidenced by the reduction in heterogeneity (I^2^ < 30% regardless of the transformation). Regarding the comparison between wearable and non-wearable systems, several studies have evaluated them, highlighting their respective advantages and limitations. Non-wearable systems provide a comprehensive representation of posture and perform particularly well when acquisition conditions are controlled for posture recognition (Dang et al., 2020), whereas wearables remain advantageous due to their robustness to environmental conditions and their ability to provide continuous tracking, despite constraints related to sensor wear and positioning (Yan et al., 2019). However, these studies do not provide a quantitative comparison of performance metrics through statistical analysis. The meta-regression did not reveal any significant differences between wearable and non-wearable sensors, with the exception of the F1 score, for which wearable systems performed better across all three transformations (*p* < 0.05). This result only suggests that wearable devices provide a better overall balance between correctly identifying hazardous postures and minimizing false detections, rather than improving a single performance metric.

#### 4.3.3 Occupational activities

This meta-regression suggests that occupational activity contributes to the variability observed across studies, although its influence appears to be less pronounced than the AI method used. Studies conducted in industrial settings generally showed the best overall performance (values ranging from 97 to 100%, with higher values using logit transformation). One possible explanation is that industrial tasks are often highly standardized and repetitive, leading to more homogeneous postural patterns that are easier for AI models to learn. Furthermore, many studies on posture recognition in industrial settings are conducted under controlled experimental conditions involving a limited set of predefined tasks, which may also contribute to the better overall performance observed in this sector (Reining et al., 2019). Similarly, studies in the construction sector frequently focus on a limited number of well-defined tasks under controlled experimental conditions, which may also facilitate posture recognition and thus explain the results—which are similar to those in the industrial sector in terms of sensitivity and precision (Yu et al., 2021; Sherafat et al., 2020). The manufacturing sector has tended to perform slightly worse than the industrial and construction sectors. This sector is similar to the industrial sector because they share similar environments: repetitive tasks, relatively stable postures, and well-organized workspaces. These performance differences can be considered modest for several reasons. First, these differences were not consistently present across the transformations applied. Second, other methodological factors (sensors, AI methods, datasets, etc.) may have influenced the performance metrics.

In contrast, models developed using data from a wide range of occupational activities (the “All” category) were exposed to significantly greater variability in workers, tasks, and environmental conditions. This variability may explain the lower overall performance observed. Indeed, such diversity increases the complexity of the classification problem and generally reduces recognition performance. However, several studies have emphasized that this variability is essential for developing AI models able to generalize to unseen subjects and real-world working conditions, rather than overfitting to a single occupational context (Napoli et al., 2024; Arrotta et al., 2025).

#### 4.3.4 Ergonomic tools

This meta-regression demonstrates that the ergonomic assessment tool is a significant source of between-study variability in the field of AI-based posture recognition. However, this effect is not consistent across metrics, and none of the assessment tools consistently outperformed all the others. The RULA method consistently achieved higher classification accuracy than OSHA and REBA. The REBA and RULA methods also achieved higher sensitivity than NIOSH or binary classifications (safe/unsafe), highlighting their greater ability to detect hazardous postures. These results show that ergonomic tools based on a detailed description of posture across multiple body segments facilitate AI learning and enable models to identify more postures that are actually at risk. This feature is particularly important in the context of WMSD prevention, as it significantly reduces the occurrence of false negatives. The APR showed the highest specificity, indicating a greater ability to correctly identify safe postures while minimizing false positives. This result can be explained by the fact that risky postures are accurately identified, and the decision boundaries are therefore often clearer than in tools that evaluate multiple ergonomic dimensions simultaneously. However, the S vs. U classification generally yielded lower results in terms of precision and F1 score. This suggests that reducing the ergonomic assessment to a simplified binary decision may result in the loss of discriminating biomechanical information useful for training the AI. In other words, simplification does not necessarily lead to better recognition.

It is important to note that ergonomic tools were not originally designed to train AI models. RULA, REBA, OWAS, NIOSH, and classifications based on APR or safe/unsafe postures were each developed to address different ergonomic objectives (upper limb assessment, whole-body analysis, load handling, binary decision-making, etc.). They are therefore based on different variables and different definitions of WMSD risk assessment. It has been shown in the literature that a supervised model learns the relationship between the data and the provided labels. It never learns the actual phenomenon, but rather the definition of the labels (Goodfellow et al., 2016). Several studies have highlighted that the quality of annotations strongly influences final performance (Song et al., 2023; Northcutt et al., 2021). Thus, the AI models learned the specific characteristics of the various ergonomic tools rather than directly the risk of WMSDs to which operators are exposed. Consequently, some of the observed performance differences reflect the quality and level of detail of the ergonomic variables and parameters used, rather than any intrinsic superiority of the AI method’s algorithms.

#### 4.3.5 Heterogeneity and level of evidence

Despite all the subgroup analyses conducted and the investigations into potential effects, heterogeneity remained high for the majority of the parameters tested. This suggests that no single parameter is responsible for the significant inter-study variability. AI studies proposing posture recognition solutions for the prevention of WMSDs differ simultaneously in the AI method used, the training and test datasets, the chosen ergonomic assessment tool, the type of sensors, the work context, and the tasks performed, which result in a wide variety of postures, this partly explains the wide variation in performance observed. Other substantial methodological differences in the development and evaluation of AI-based posture recognition systems may partly account for the very high inter-study heterogeneity observed. The validation strategy can significantly influence reported performance. Conventional “k-fold” cross-validation may allow observations from the same subjects to appear in both the training and test sets, potentially leading to subject-related information leakage and optimistic performance estimates. Conversely, Leave-One-Subject-Out (LOSO) validation evaluates the model on subjects completely excluded from training and thus provides a more rigorous assessment of cross-subject generalizability. Previous studies on human activity recognition have demonstrated a substantial 13% drop in accuracy when switching from conventional “k-fold” validation to subject-independent LOSO validation (Bragança et al., 2022). Variability in feature extraction and selection strategies is another factor that may contribute to the significant heterogeneity observed across studies. Both the nature and the number of input features can have a considerable impact on recognition performance (Chong et al., 2021; Capela et al., 2015). Finally, differences in prevalence and class definitions can directly affect performance metrics. Imbalanced datasets can yield an apparently high overall accuracy while masking poor recognition of minority classes, whereas differences in the number and definition of ergonomic risk categories alter the complexity of the classification task as well as the resulting confusion matrix. This issue is particularly relevant in the present review, as the included studies used heterogeneous frameworks, ranging from binary classifications (safe/unsafe) to approaches based on the RULA, REBA, OWAS, or NIOSH methods, or on the identification of awkward postures. Overall, all these differences provide plausible methodological explanations for the significant residual heterogeneity observed across studies despite the subgroup analyses conducted. Substantial residual heterogeneity may also reflect interactions and confounding factors among methodological factors. For example, AI methodology and sensor type may not be independent, as certain AI approaches may be associated with specific detection modalities. Although these factors were examined separately in the subgroup analyses and meta-regressions presented here, their respective contributions cannot be fully disentangled. Future studies involving a larger number of independent studies could therefore use a multivariate meta-regression to simultaneously account for several relevant moderators. Furthermore, hierarchical meta-analytic models could account for the dependence among multiple performance estimates from the same study. Such approaches would make it possible to determine the independent contribution of each methodological factor and to identify potential interactions, while explaining a larger proportion of the observed heterogeneity across studies. However, given the limited number of included studies and the large number of potential moderators, these analyses were not conducted to avoid overparameterization of the model and instability in the estimates.

Furthermore, the effects observed among the various parameters identified by the pairwise mixed-effects meta-regressions must be interpreted with caution, given the very low level of confidence assigned when evaluating the results using the GRADE approach. This finding clearly demonstrates the need to establish a strict and robust methodological framework when proposing new AI tools. This will provide access to more detailed and rigorous information, including the involvement of experts performing professional activities under real-world conditions, comprehensive validation of the algorithms, and the number and nature of the features used by the models for posture recognition, etc.

### 4.4 Practical outcomes and future research directions

Despite relatively high posture detection performance (>85%) and an examination of the effects of various parameters, the full implementation of the PRISMA methodology in this meta-analysis revealed a high risk of bias in the included studies and, consequently, a very low level of confidence in the reported results. Thus, as things stand, the deployment of existing solutions in real-world settings remains limited. This calls for caution and underscores the need for additional, more rigorous studies to confirm these results and ensure the transferability of prevention tools to the workplace. A key area for improvement concerns the characteristics of the subjects used for training and validating AI models. The sample size must be large, with the expected level of expertise, and with diverse anthropometric and demographic characteristics to ensure easier generalization of the results when an unknown subject is encountered (Arrotta et al., 2025). The tasks and postures used during the training phase must be as close as possible to real-world work situations in order to ensure the model’s performance when deployed in the work environment. The second focus area concerns the validation procedures for the AI model to ensure its viability. For internal validation—unlike a traditional k-cross-fold validation, in which the model’s ability to recognize new postures in a subject already encountered in the training dataset is tested—the use of LOSO appears preferable, as it tests the model’s ability to function in the presence of a new subject. It therefore allows for greater generalization of the results, even if the performance obtained is generally lower (Wang et al., 2019). External validation, which is very rarely performed in the existing literature, must also be conducted. It is considered the most rigorous strategy for evaluating the practical applicability of AI models, as it measures their ability to generalize their results to independent populations and acquisition conditions, rather than to data from the training dataset (Collins et al., 2015). This aspect is particularly important in the field of AI-based posture recognition for the prevention of work-related musculoskeletal disorders (WMSDs), as workers, occupational tasks, workplaces, and acquisition conditions may vary considerably across professional environments. Consequently, models evaluated exclusively through internal validation procedures often produce overly optimistic performance estimates. When deployed under real-world conditions, their performance dropped to 50% or even 25% (Palmerini et al., 2020). A third avenue for improvement concerns the substantial between-study heterogeneity observed in the present meta-analysis. Numerous factors influence the performance of AI-based posture recognition models, including both dataset-related characteristics and intrinsic model properties. Since the dataset has a direct impact on model performance (Althnian et al., 2021), the use of common benchmark datasets would provide a standardized basis for objectively comparing the performance of different AI approaches (Gorce and Jacquier-Bret, 2026b).

From a practical perspective, future research should continue to prioritize improvements in model sensitivity to ensure that all hazardous postures associated with the development of WMSDs are correctly identified, while simultaneously adhering to methodological recommendations that promote robust generalization and reliable deployment under real-world working conditions.

### 4.5 Limitations

In the various included studies, the authors tested different tasks in different postures. The proposed subgroup analysis considered only differences between occupational activities and did not explore these differences in tasks and postures. The variability of these two parameters was not taken into account, and the studies were grouped together. Future studies could be conducted to specifically examine their effects on performance.

The second limitation concerns the conditions under which the performance of posture detection systems was evaluated. The datasets were highly heterogeneous: in addition to the tasks and postures, which differed across studies, the number of subjects, their demographic and anthropometric characteristics, and the number and nature of the features used also varied. This has a direct impact on the training phase of AI methods and, consequently, on the evaluation of the performance of the proposed solutions. It therefore seems important and necessary to standardize the evaluation conditions in order to accurately and objectively compare posture detection solutions.

Another limitation relates to the fact that several of the included studies reported multiple performance estimates derived from different AI algorithms or configurations, evaluated on the same participants and/or datasets, and that all of these were included in the meta-analysis. As a result, some effect sizes were not entirely independent. In the absence of explicit modeling of intra-study correlations using robust variance estimation (RVE) or multilevel random-effects models, the pooled performance may have been affected by a disproportionate influence from these studies that had multiple values. Thus, future meta-analyses with sufficient data should explicitly account for correlated effect sizes by using RVE or multilevel meta-analytic models. Another approach would be to retain only the best algorithm from each study for the meta-analysis.

The analysis of possible causes of heterogeneity could be expanded. It has been shown that, beyond the chosen AI method, algorithms also have an effect on performance levels. It would therefore be useful to deepen the analysis by pooling and comparing performance results by algorithm type (SVM, KNN, RF, DT, CNN, LSTM, etc.).

Another limitation was related to the amount of data available depending on the various parameters considered. Indeed, some subgroup analyses could not be conducted due to a lack of data (e.g., occupational activity in sports and agriculture, or specificity by sensor type) or included a small number of data (<10). Further studies are therefore needed to strengthen the observed results.

Finally, some limitations are methodological in nature. They relate to the inclusion and exclusion criteria used during the selection phase. The combination of keywords, the choice of datasets, and the decision to include only original, peer-reviewed studies written in English may have led to the omission of certain studies that could have enriched the systematic review.

## 5 Conclusion

This systematic review and meta-analysis demonstrated the high performance of AI-based posture recognition systems for the assessment of WMSD risk. Although the choice of proportion transformation did not influence the overall scientific conclusions, as pooled estimates and between-study heterogeneity remained largely unchanged, the logit transformation consistently improved the statistical properties of the data, particularly in terms of distributional symmetry and conformity with the normality assumption. Further analyses revealed that the AI approach, type of sensors, occupational activity, and ergonomic risk assessment tool all significantly influenced the reported performance metrics. These findings were obtained following the PRISMA guidelines, including a comprehensive assessment of the risk of bias. However, the risk-of-bias analyses indicated that the overall certainty of the evidence remained very low, and the findings should therefore be interpreted with caution, and prudence is required regarding the deployment of the systems under real-world conditions. Nevertheless, the present study provides valuable evidence for researchers, practitioners, and policy-makers by identifying methodological factors associated with AI performance in posture recognition. These findings may contribute to the development of more accurate, robust, and generalizable AI systems for the prevention of WMSDs, ultimately supporting improvements in occupational health and worker safety.

## Data Availability

The original contributions presented in the study are included in the article/Supplementary material, further inquiries can be directed to the corresponding author.

## Supplementary material

SUPPLEMENTARY FIGURE S1 Forest plot of logit-transformed accuracy pooled across all studies. Each square represents the logit-transformed accuracy of one algorithm. The horizontal bars represent the 95% confidence interval. The diamond at the bottom represents the overall pooled accuracy.

SUPPLEMENTARY FIGURE S2 Forest plot of logit-transformed specificity pooled across all studies. Each square represents the logit-transformed specificity of one algorithm. The horizontal bars represent the 95% confidence interval. The diamond at the bottom represents the overall pooled specificity.

SUPPLEMENTARY FIGURE S3 Forest plot of logit-transformed sensitivity pooled across all studies. Each square represents the logit-transformed sensitivity of one algorithm. The horizontal bars represent the 95% confidence interval. The diamond at the bottom represents the overall pooled sensitivity.

SUPPLEMENTARY FIGURE S4 Forest plot of logit-transformed precision pooled across all studies. Each square represents the logit-transformed precision of one algorithm. The horizontal bars represent the 95% confidence interval. The diamond at the bottom represents the overall pooled precision.

SUPPLEMENTARY FIGURE S5 Forest plot of logit-transformed F1 score pooled across all studies. Each square represents the logit-transformed F1 score of one algorithm. The horizontal bars represent the 95% confidence interval. The diamond at the bottom represents the overall pooled F1 score.

SUPPLEMENTARY FIGURE S6 Funnel plots and corresponding Egger’s test results for each performance parameter using untransformed values.

SUPPLEMENTARY FIGURE S7 Funnel plots and corresponding Egger’s test results for each performance parameter using Freeman-Tukey transformation.
