# Supplementary material for: Meta-analysis of AI posture recognition for work-related musculoskeletal disorders prevention in occupational activities: comparison between untransformed, logit and Freeman-Tukey transformed performance
Source: Front Artif Intell. 2026 Oct 7;9:1933096. doi: 10.3389/frai.2026.1933096 (PMC13683794; doi:10.3389/frai.2026.1933096)
Supplement: Supplementary file 1 [file Table_1.docx]

**Table S1.** Sensor category (wearable, non-wearable, hybrid) comparison of pooled values and heterogeneity parameters obtained with each transformation for the five performance parameters.

| **Parameter** | **Transformation** | **Comparison** | **β (95% CI)^a^** | **z** | **p(Wald)** | **QM** | **p(QM)** |
| --- | --- | --- | --- | --- | --- | --- | --- |
| Accuracy | Untransformed | NW vs W | -0.026 (-0.076−0.024) | -1.005 | 0.315 | 1.011 | 0.315 |
|  |  | H vs W | -0.037 (-0.082−0.008) | -1.605 | 0.109 | 2.576 | 0.109 |
|  |  | H vs NW | -0.010 (-0.072−0.052) | -0.320 | 0.749 | 0.102 | 0.749 |
|  | Logit | NW vs W | -0.537 (-0.989−-0.085) | -2.327 | 0.020* | 5.413 | 0.020 |
|  |  | H vs W | -0.259 (-0.679−0.160) | -1.211 | 0.226 | 1.465 | 0.226 |
|  |  | H vs NW | 0.292 (-0.238−0.821) | 1.080 | 0.280 | 1.166 | 0.280 |
|  | Freeman-Tukey | NW vs W | -0.064 (-0.134−0.007) | -1.774 | 0.076 | 3.149 | 0.076 |
|  |  | H vs W | -0.051 (-0.113−0.012) | -1.587 | 0.113 | 2.519 | 0.113 |
|  |  | H vs NW | 0.014 (-0.068−0.095) | 0.325 | 0.745 | 0.106 | 0.745 |
| Sensitivity | Untransformed | NW vs W | 0.024 (-0.043−0.090) | 0.697 | 0.486 | 0.485 | 0.486 |
|  |  | H vs W | 0.146 (-0.002−0.294) | 1.939 | 0.053 | 3.761 | 0.053 |
|  |  | H vs NW | 0.116 (0.023−0.210) | 2.433 | 0.015* | 5.920 | 0.015 |
|  | Logit | NW vs W | 0.336 (-0.272−0.943) | 1.083 | 0.279 | 1.174 | 0.279 |
|  |  | H vs W | 3.055 (1.797−4.313) | 4.761 | 0.000* | 22.662 | 0.000 |
|  |  | H vs NW | 2.740 (1.472−4.008) | 4.235 | 0.000* | 17.934 | 0.000 |
|  | Freeman-Tukey | NW vs W | 0.044 (-0.049−0.137) | 0.923 | 0.356 | 0.852 | 0.356 |
|  |  | H vs W | 0.282 (0.081−0.483) | 2.746 | 0.006* | 7.540 | 0.006 |
|  |  | H vs NW | 0.235 (0.079−0.391) | 2.947 | 0.003* | 8.687 | 0.003 |
| Precision | Untransformed | NW vs W | -0.009 (-0.071−0.053) | -0.286 | 0.775 | 0.082 | 0.775 |
|  |  | H vs W | 0.128 (0.003−0.253) | 2.011 | 0.044* | 4.042 | 0.044 |
|  |  | H vs NW | 0.135 (0.026−0.244) | 2.430 | 0.015* | 5.904 | 0.015 |
|  | Logit | NW vs W | -0.042 (-0.698−0.614) | -0.126 | 0.900 | 0.016 | 0.900 |
|  |  | H vs W | 2.813 (1.526−4.100) | 4.285 | 0.000* | 18.362 | 0.000 |
|  |  | H vs NW | 2.880 (1.593−4.167) | 4.387 | 0.000* | 19.242 | 0.000 |
|  | Freeman-Tukey | NW vs W | -0.003 (-0.092−0.087) | -0.061 | 0.951 | 0.004 | 0.951 |
|  |  | H vs W | 0.256 (0.080−0.433) | 2.843 | 0.005* | 8.082 | 0.005 |
|  |  | H vs NW | 0.258 (0.091−0.425) | 3.022 | 0.003* | 9.133 | 0.003 |
| F1 score | Untransformed | NW vs W | -0.069 (-0.111−-0.027) | -3.236 | 0.001* | 10.470 | 0.001 |
|  |  | H vs W | -0.058 (-0.109−-0.007) | -2.218 | 0.027* | 4.920 | 0.027 |
|  |  | H vs NW | 0.013 (-0.046−0.073) | 0.435 | 0.663 | 0.189 | 0.663 |
|  | Logit | NW vs W | -0.728 (-1.174−-0.283) | -3.207 | 0.001* | 10.283 | 0.001 |
|  |  | H vs W | -0.166 (-0.846−0.514) | -0.479 | 0.632 | 0.229 | 0.632 |
|  |  | H vs NW | 0.550 (-0.087−1.187) | 1.694 | 0.090 | 2.868 | 0.090 |
|  | Freeman-Tukey | NW vs W | -0.105 (-0.171−-0.040) | -3.170 | 0.002* | 10.051 | 0.002 |
|  |  | H vs W | -0.068 (-0.149−0.014) | -1.616 | 0.106 | 2.610 | 0.106 |
|  |  | H vs NW | 0.039 (-0.049- 0.127) | 0.878 | 0.380 | 0.770 | 0.380 |

^a^: The **β** values were reported as proportions for the untransformed data, on a logit scale for the logit transformation, and as raw values for the Freeman-Tukey transformation.; * indicates a significant difference. 95% CI: 95% confidence interval; W: wearable sensors; NW: non-wearable sensors; H: hybrid solution.
