# Supplementary material for: Meta-analysis of AI posture recognition for work-related musculoskeletal disorders prevention in occupational activities: comparison between untransformed, logit and Freeman-Tukey transformed performance
Source: Front Artif Intell. 2026 Oct 7;9:1933096. doi: 10.3389/frai.2026.1933096 (PMC13683794; doi:10.3389/frai.2026.1933096)
Supplement: Supplementary file 2 [file Table_2.docx]

**Table S2.** Occupational activity (All, construction, industry, manufacturing) comparison of pooled values and heterogeneity parameters obtained with each transformation for the five performance parameters.

| **Parameter** | **Transformation** | **Comparison** | **β (95% CI)^a^** | **z** | **P(Wald)** | **QM** | **p(QM)** |
| --- | --- | --- | --- | --- | --- | --- | --- |
| Accuracy | Untransformed | Const. vs All | -0.040 (-0.297−0.217) | -0.307 | 0.759 | 0.094 | 0.759 |
|  |  | Ind. vs All | 0.094 (0.060−0.129) | 5.375 | 0.000* | 28.892 | 0.000 |
|  |  | Manuf. vs All | 0.009 (-0.162−0.180) | 0.106 | 0.916 | 0.011 | 0.916 |
|  |  | Ind. vs Const. | 0.162 (0.037−0.286) | 2.544 | 0.011* | 6.473 | 0.011 |
|  |  | Manuf. vs Const. | 0.052 (0.012−0.092) | 2.523 | 0.012* | 6.367 | 0.012 |
|  |  | Manuf. vs Ind. | -0.109 (-0.189−-0.029) | -2.668 | 0.008* | 7.118 | 0.008 |
|  | Logit | Const. vs All | 0.159 (-1.802−2.119) | 0.159 | 0.874 | 0.025 | 0.874 |
|  |  | Ind. vs All | 1.634 (0.726−2.543) | 3.525 | 0.000* | 12.425 | 0.000 |
|  |  | Manuf. vs All | 0.528 (-1.324−2.381) | 0.559 | 0.576 | 0.312 | 0.576 |
|  |  | Ind. vs Const. | 1.972 (0.852−3.092) | 3.452 | 0.001* | 11.917 | 0.001 |
|  |  | Manuf. vs Const. | 0.376 (0.005−0.748) | 1.985 | 0.047* | 3.939 | 0.047 |
|  |  | Manuf. vs Ind. | -1.579 (-2.641−-0.517) | -2.913 | 0.004* | 8.487 | 0.004 |
|  | Freeman-Tukey | Const. vs All | -0.014 (-0.341−0.314) | -0.082 | 0.935 | 0.007 | 0.935 |
|  |  | Ind. vs All | 0.238 (0.095−0.380) | 3.263 | 0.001* | 10.647 | 0.001 |
|  |  | Manuf. vs All | 0.049 (-0.212−0.310) | 0.365 | 0.715 | 0.134 | 0.715 |
|  |  | Ind. vs Const. | 0.270 (0.109−0.432) | 3.281 | 0.001* | 10.767 | 0.001 |
|  |  | Manuf. vs Const. | 0.065 (0.009−0.120) | 2.283 | 0.023* | 5.211 | 0.023 |
|  |  | Manuf. vs Ind. | -0.205 (-0.333−-0.078) | -3.159 | 0.002* | 9.982 | 0.002 |
| Specificity | Untransformed | Manuf. vs Const. | -0.163 (-0.257−-0.069) | -3.395 | 0.001* | 11.526 | 0.001 |
|  | Logit | Manuf. vs Const. | -3.583 (-4.352−-2.814) | -9.133 | 0.000* | 83.416 | 0.000 |
|  | Freeman-Tukey | Manuf. vs Const. | -0.318 (-0.442−-0.193) | -5.008 | 0.000* | 25.077 | 0.000 |
| Sensitivity | Untransformed | Const. vs All | 0.049 (-0.009−0.107) | 1.654 | 0.098 | 2.735 | 0.098 |
|  |  | Ind. vs All | 0.085 (-0.002−0.173) | 1.909 | 0.056 | 3.643 | 0.056 |
|  |  | Manuf. vs All | -0.054 (-0.147−0.038) | -1.149 | 0.251 | 1.321 | 0.251 |
|  |  | Ind. vs Const. | 0.036 (-0.015−0.087) | 1.397 | 0.163 | 1.951 | 0.163 |
|  |  | Manuf. vs Const. | -0.104 (-0.196−-0.012) | -2.219 | 0.027* | 4.924 | 0.027 |
|  |  | Manuf. vs Ind. | -0.140 (-0.295−0.014) | -1.779 | 0.075 | 3.164 | 0.075 |
|  | Logit | Const. vs All | 0.644 (-0.120−1.407) | 1.653 | 0.098 | 2.731 | 0.098 |
|  |  | Ind. vs All | 1.614 (0.298−2.929) | 2.404 | 0.016* | 5.781 | 0.016 |
|  |  | Manuf. vs All | -0.424 (-1.290−0.441) | -0.961 | 0.336 | 0.924 | 0.336 |
|  |  | Ind. vs Const. | 0.894 (-0.257−2.045) | 1.523 | 0.128 | 2.319 | 0.128 |
|  |  | Manuf. vs Const. | -1.033 (-1.891−-0.175) | -2.358 | 0.018* | 5.562 | 0.018 |
|  |  | Manuf. vs Ind. | -2.130 (-3.717−-0.543) | -2.631 | 0.009* | 6.921 | 0.009 |
|  | Freeman-Tukey | Const. vs All | 0.081 (-0.016−0.177) | 1.644 | 0.100 | 2.703 | 0.100 |
|  |  | Ind. vs All | 0.176 (0.033−0.320) | 2.406 | 0.016* | 5.791 | 0.016 |
|  |  | Manuf. vs All | -0.080 (-0.208−0.048) | -1.224 | 0.221 | 1.498 | 0.221 |
|  |  | Ind. vs Const. | 0.096 (-0.008−0.200) | 1.817 | 0.069 | 3.301 | 0.069 |
|  |  | Manuf. vs Const. | -0.161 (-0.288−-0.034) | -2.482 | 0.013* | 6.162 | 0.013 |
|  |  | Manuf. vs Ind. | -0.256 (-0.470−-0.043) | -2.354 | 0.019* | 5.543 | 0.019 |
| Precision | Untransformed | Const. vs All | 0.105 (0.034−0.176) | 2.911 | 0.004* | 8.473 | 0.004 |
|  |  | Ind. vs All | 0.116 (-0.002−0.233) | 1.931 | 0.054 | 3.727 | 0.054 |
|  |  | Manuf. vs All | 0.001 -0.085−0.086) | 0.018 | 0.986 | 0.000 | 0.986 |
|  |  | Ind. vs Const. | 0.013 (-0.018−0.043) | 0.822 | 0.411 | 0.675 | 0.411 |
|  |  | Manuf. vs Const. | -0.104 (-0.184−-0.024) | -2.552 | 0.011* | 6.515 | 0.011 |
|  |  | Manuf. vs Ind. | -0.115 (-0.248−0.018) | -1.693 | 0.091 | 2.865 | 0.091 |
|  | Logit | Const. vs All | 1.532 (0.609−2.454) | 3.254 | 0.001* | 10.586 | 0.001 |
|  |  | Ind. vs All | 1.573 (0.239−2.907) | 2.312 | 0.021* | 5.343 | 0.021 |
|  |  | Manuf. vs All | 0.130 (-0.754−1.014) | 0.288 | 0.774 | 0.083 | 0.774 |
|  |  | Ind. vs Const. | 0.024 (-1.274−1.322) | 0.036 | 0.971 | 0.001 | 0.971 |
|  |  | Manuf. vs Const. | -1.372 (-2.271−-0.474) | -2.994 | 0.003* | 8.962 | 0.003 |
|  |  | Manuf. vs Ind. | -1.461 (-2.958−0.036) | -1.913 | 0.056 | 3.659 | 0.056 |
|  | Freeman-Tukey | Const. vs All | 0.172 (0.064−0.280) | 3.120 | 0.002* | 9.731 | 0.002 |
|  |  | Ind. vs All | 0.195 (0.024−0.367) | 2.232 | 0.026* | 4.980 | 0.026 |
|  |  | Manuf. vs All | -0.002 (-0.124−0.119) | -0.035 | 0.972 | 0.001 | 0.972 |
|  |  | Ind. vs Const. | 0.025 (-0.066−0.115) | 0.529 | 0.597 | 0.280 | 0.597 |
|  |  | Manuf. vs Const. | -0.173 (-0.289−-0.058) | -2.932 | 0.003* | 8.596 | 0.003 |
|  |  | Manuf. vs Ind. | -0.198 (-0.389−-0.006) | -2.022 | 0.043* | 4.088 | 0.043 |
| F1 score | Untransformed | Const. vs All | 0.008 (-0.054−0.071) | 0.264 | 0.792 | 0.070 | 0.792 |
|  |  | Ind. vs All | 0.103 (0.006−0.200) | 2.077 | 0.038* | 4.312 | 0.038 |
|  |  | Manuf. vs All | 0.030 (-0.024−0.084) | 1.078 | 0.281 | 1.162 | 0.281 |
|  |  | Ind. vs Const. | 0.095 (0.008−0.181) | 2.140 | 0.032* | 4.578 | 0.032 |
|  |  | Manuf. vs Const. | 0.022 (-0.022−0.065) | 0.982 | 0.326 | 0.964 | 0.326 |
|  |  | Manuf. vs Ind. | -0.073 (-0.144−-0.003) | -2.036 | 0.042* | 4.144 | 0.042 |
|  | Logit | Const. vs All | 0.088 (-0.546−0.722) | 0.272 | 0.786 | 0.074 | 0.786 |
|  |  | Ind. vs All | 1.451 (0.339−2.562) | 2.559 | 0.011* | 6.546 | 0.011 |
|  |  | Manuf. vs All | 0.377 (-0.312−1.067) | 1.072 | 0.284 | 1.149 | 0.284 |
|  |  | Ind. vs Const. | 1.367 (0.293−2.441) | 2.495 | 0.013* | 6.223 | 0.013 |
|  |  | Manuf. vs Const. | 0.300 (-0.232−0.831) | 1.106 | 0.269 | 1.223 | 0.269 |
|  |  | Manuf. vs Ind. | -1.107 (-2.286−0.073) | -1.839 | 0.066 | 3.382 | 0.066 |
|  | Freeman-Tukey | Const. vs All | 0.007 (-0.085−0.098) | 0.140 | 0.889 | 0.020 | 0.889 |
|  |  | Ind. vs All | 0.185 (0.037−0.333) | 2.444 | 0.015* | 5.972 | 0.015 |
|  |  | Manuf. vs All | 0.042 (-0.044−0.128) | 0.959 | 0.338 | 0.919 | 0.338 |
|  |  | Ind. vs Const. | 0.179 (0.048−0.309) | 2.676 | 0.007* | 7.163 | 0.007 |
|  |  | Manuf. vs Const. | 0.036 (-0.031−0.102) | 1.040 | 0.298 | 1.082 | 0.298 |
|  |  | Manuf. vs Ind. | -0.143 (-0.265−-0.022) | -2.310 | 0.021* | 5.337 | 0.021 |

^a^: The **β** values were reported as proportions for the untransformed data, on a logit scale for the logit transformation, and as raw values for the Freeman-Tukey transformation.; * indicates a significant difference. 95% CI: 95% confidence interval; Const.: construction; Ind.: industry; Manuf.: Manufacturing.
