# Supplementary material for: Meta-analysis of AI posture recognition for work-related musculoskeletal disorders prevention in occupational activities: comparison between untransformed, logit and Freeman-Tukey transformed performance
Source: Front Artif Intell. 2026 Oct 7;9:1933096. doi: 10.3389/frai.2026.1933096 (PMC13683794; doi:10.3389/frai.2026.1933096)
Supplement: Supplementary file 4 [file Table_4.docx]

**Table S4.** Detailed GRADE analysis for FT transformation.

| **Performance parameter** | **Number of Studies** | **Certainty Assessment** | | | | | | **Effect** | | | **Overall level of evidence** |
| --- | --- | --- | --- | --- | --- | --- | --- | --- | --- | --- | --- |
|  |  | **Study  Design** | **Publication Bias  (Egger’s Test)** | **Indirectness ^a^** | **Inconsistency ^b^** | **Imprecision ^c^** | **Risk of Bias ^d^** | ***n*** | **Event Rate** | **(95% CI)** |  |
| Accuracy | 21 | DTAS | Not serious  (p = 0.228) | Serious | Serious  (I² = 100%) | Not serious | Serious | 241 | 86.9% | 85.1−88.6% | Very low  ⬤◯◯◯ |
| Specificity | 4 | DTAS | Serious  (p < 0.001) | Serious | Serious  (I² = 100%) | Not serious | Serious | 98 | 88.3% | 84.7−91.4% | Very low  ⬤◯◯◯ |
| Sensitivity | 15 | DTAS | Serious  (p < 0.001) | Serious | Serious  (I² = 100%) | Not serious | Serious | 167 | 89.6% | 86.9−92.1% | Very low  ⬤◯◯◯ |
| Precision | 14 | DTAS | Serious  (p < 0.001) | Serious | Serious  (I² = 100%) | Not serious | Serious | 139 | 90.5% | 87.9−92.8% | Very low  ⬤◯◯◯ |
| F1 score | 18 | DTAS | Not serious  (p = 0.051) | Serious | Serious  (I² = 99.9%) | Not serious | Serious | 148 | 90.1% | 88.1−91.9% | Very low  ⬤◯◯◯ |

DTAS: Diagnostic test accuracy study; n: number of available data; 95% CI: 95% confidence interval; *n*: number of available data.

GRADE overall quality significance: ⬤◯◯◯ = Very low; ⬤⬤◯◯ = Low; ⬤⬤⬤◯ = Moderate; ⬤⬤⬤⬤ = High.

^a^ Studied population correspond to the population in study

^b^ Serious if I² > 50%

^c^ Serious if 95% CI Range Difference > 50% of Event Rate

^d^ Studies have at least a fair critical appraisal score
